# Comparative Analyses of Phyllosphere Bacterial Communities and Metabolomes in Newly Developed Needles of *Cunninghamia lanceolata* (Lamb.) Hook. at Four Stages of Stand Growth

**DOI:** 10.3389/fpls.2021.717643

**Published:** 2021-09-28

**Authors:** Kun Sun, Honggang Sun, Zonghao Qiu, Qiang Liu

**Affiliations:** ^1^Key Laboratory of Subtropical Siviculture of State Forestry and Grassland Administration, Research Institute of Subtropical Forestry of Chinese Academy of Forestry, Hangzhou, China; ^2^Department of Tree Genetics, College of Forestry, Beihua University, Jilin, China; ^3^Laboratory of Molecular Biology, Institute of Biochemistry and Molecular Biology, Albert-Ludwigs-Universität Freiburg, Freiburg im Breisgau, Germany; ^4^Department of Plant Sciences, School of Life Sciences, Jilin Normal University, Siping, China

**Keywords:** *Cunninghamia lanceolata*, phyllosphere bacterial community, metabolic profile, host-bacteria interaction, functional genes

## Abstract

Host-plant-associated bacteria affect the growth, vigor, and nutrient availability of the host plant. However, phyllosphere bacteria have received less research attention and their functions remain elusive, especially in forest ecosystems. In this study, we collected newly developed needles from sapling (age 5 years), juvenile (15 years), mature (25 years), and overmature (35 years) stands of Chinese fir [*Cunninghamia lanceolata* (Lamb.) Hook]. We analyzed changes in phyllosphere bacterial communities, their functional genes, and metabolic activity among different stand ages. The results showed that phyllosphere bacterial communities changed, both in relative abundance and in composition, with an increase in stand age. Community abundance predominantly changed in the orders Campylobacterales, Pseudonocardiales, Deinococcales, Gemmatimonadales, Betaproteobacteriales, Chthoniobacterales, and Propionibacteriales. Functional predictions indicated the genes of microbial communities for carbon metabolism, nitrogen metabolism, antibiotic biosynthesis, flavonoids biosynthesis, and steroid hormone biosynthesis varied; some bacteria were strongly correlated with some metabolites. A total of 112 differential metabolites, including lipids, benzenoids, and flavonoids, were identified. Trigonelline, proline, leucine, and phenylalanine concentrations increased with stand age. Flavonoids concentrations were higher in sapling stands than in other stands, but the transcript levels of genes associated with flavonoids biosynthesis in the newly developed needles of saplings were lower than those of other stands. The nutritional requirements and competition between individual trees at different growth stages shaped the phyllosphere bacterial community and host–bacteria interaction. Gene expression related to the secondary metabolism of shikimate, mevalonate, terpenoids, tocopherol, phenylpropanoids, phenols, alkaloids, carotenoids, betains, wax, and flavonoids pathways were clearly different in Chinese fir at different ages. This study provides an overview of phyllosphere bacteria, metabolism, and transcriptome in Chinese fir of different stand ages and highlights the value of an integrated approach to understand the molecular mechanisms associated with biosynthesis.

## Introduction

Microbial communities associated with host plants in natural ecosystems are often considered to be an extension of the phenotypes of their host plants. These extended phenotypes are predominantly affected by host traits, especially the chemical composition of host tissues, and environmental variables, such as temperature and precipitation (Helander et al., 1993; Ahlholm et al., 2002; Chareprasert et al., 2006; Verma et al., 2014). Bacteria are important components of the microbial communities associated with host plants. Host-plant-associated bacteria affect the growth, health, and nutrient absorption and cycling of the host plant, especially plant growth-promoting rhizobacteria (Chen et al., 2020; Swarnalakshmi et al., 2020). Previous studies of forest bacterial communities have focused on belowground processes, such as carbon sequestration, root activity, and litter decomposition (Prada-Salcedo et al., 2020; Truu et al., 2020; Yokobe et al., 2020). Little attention has been paid to the functions of foliar bacterial communities in forest ecosystems.

The total leaf surface area greatly exceeds the terrestrial land area in forest ecosystems. Therefore, plant leaves and needles provide vast habitats for diverse bacteria and fungi, especially foliar endophytic fungi (Saucedo-Garcia et al., 2014; Jia et al., 2020; Quiring et al., 2020; Shahrtash and Brown, 2020) and bacteria (Rakotoniriana et al., 2013; Yu et al., 2015; Carrell et al., 2016). Foliar endophytes perform multiple functions (Jia et al., 2020) and participate in nutrient uptake (Madhaiyan et al., 2015; Moyes et al., 2016; Christian et al., 2019). Bacteria that inhabit the leaf surface (or phyllosphere) have received less research attention than foliar endophytic bacteria and rhizosphere bacteria (Baldrian, 2017). Furthermore, the ecological functions of phyllosphere bacteria remain elusive.

Compared with foliar endophytic bacteria, phyllosphere bacterial communities are more readily affected by environmental factors because they are exposed to a constantly changing environment, especially to dynamic changes in solar irradiance, temperature, and moisture. Thus, the phyllosphere typically exhibits lower bacterial diversity and abundance in comparison to bacterial communities in the rhizosphere (Bringel and Couée, 2015). A typical phyllosphere bacterial community may comprise ~10^6^-10^7^ bacterial cells within a leaf surface area of 1 cm^2^ (Bulgarelli et al., 2013). Owing to the extensive bacterial gene pools and functional redundancy, the bacteria that colonize the phyllosphere influence the host plant irrespective of the community composition, for example, by modifying the nitrogen cycle, plant hormone production, secretion of biosurfactants, and host resistance to abiotic and biotic stress (Knief et al., 2010; Burch et al., 2014; Rico et al., 2014). Phyllosphere bacteria also influence leaf litter decomposition because native species compete for ecological niches through the depletion of nutrient pools and the production of antibiotic molecules (Creamer et al., 2015; Ritpitakphong et al., 2016). Bacterial communities are subject to diverse selective factors, including host resistance, host age, the phyllosphere nutrient environment, soil types, and climate conditions (Lindström and Langenheder, 2012; Williams et al., 2013).

In forest ecosystems, inter- and intra-specific competition gradually increase with stand age. Competition strengthens with an increase in canopy density, and consequently changes the crown structure and phyllosphere environment of individual trees (Zhang et al., 2020). In climax and subclimax forest communities, the crown structures and environment remain relatively stable, thus the phyllosphere bacterial communities of dominant trees are also relatively stable. However, as an individual tree grows, the phyllosphere bacterial community must respond to continual changes in the phyllosphere environment and foliar nutrient supply. To date, the influence of tree growth on phyllosphere bacterial communities has not been well-studied.

Deciduous trees replace their leaves annually, thus the foliar microbiome of deciduous trees is more dynamic than that of evergreen broad-leaved trees and conifers (Augusto et al., 2015). In evergreen conifers, the chemical composition of needles changes with an increase in leaf age. Leaf chemical composition is shaped by the host trees and phyllosphere microbes, and strongly influences litter quality, nutrient cycles, and the succession potential of a community. However, previous studies have largely focused on the chemical composition of leaf litter rather than that of fresh leaves and needles (Tláskal et al., 2016; Wang W. B. et al., 2019).

Chinese fir, a typical evergreen conifer species, is the most important commercial forest tree grown in subtropical areas of China. Plantations comprise 11.39 million ha planted in pure or mixed stands with a stocking volume of 8.52 billion m^3^ (State Forestry Administration of China, 2019). In subtropical areas, a minimum cutting diameter of 15 cm was determined for Chinese fir at a rotation age of at least 16 years (Zhou H. et al., 2016). Deterioration in soil fertility reduces the growth of Chinese fir in continuous plantations (Chen et al., 2004). However, improvement in the growth of Chinese fir plantations is critical to maintain sustainable timber production in China. In this study, we investigated changes in the phyllosphere bacterial community and metabolome of newly developed needles of Chinese fir trees from sapling, juvenile, mature, and overmature stands (age 5, 15, 25, and 35 years, respectively) based on stand growth trajectory (Liu and Tong, 1980). In addition, the molecular mechanisms associated with secondary metabolism biosynthesis were investigated using the transcriptome. The aim was to further explore the relationship between the phyllosphere bacterial communities and metabolite profiles with increasing tree age.

## Materials and Methods

### Experimental Sites

The study sites were located at the Fengshushan Forestry Farm (29°11′ N, 117°32′ E), Jiangxi province, China. The study area has a subtropical monsoon climate. The mean annual temperature, mean temperature in January, and mean temperature in July are 17.1, 4.6, and 28.7°C, respectively. The extreme high and low temperatures are 41.8°C (recorded on 29 August 1967) and −10.9°C (13 January 1963). Total radiation is 109.8 Kcal•cm^−2^ and mean annual insolation duration is 2009.5 h. The mean annual precipitation is 1763.5 mm. The predominant wind direction is northeasterly. The frost-free season is 248 d. The forest soil type is a red-yellow soil, and the average soil depth is ca. 40 cm. Information on soil properties, including the concentration of total N, ammonium N, total P, available P, total K, available K, and soil organic matter, are detailed in Table 1. Associated small shrubs and perennial herbs growing in the Chinese fir plantations include *Loropetalum chinense, Eurya hebeclados, Premna microphylla, Maesa japonica, Miscanthus floridulus, Viola diffusa, Erigeron annuus*, and *Cibotium barometz*.

**Table 1:** Stand and site information of sapling (5-), juvenile (15-), mature (25-), and overmature (35-years old) Chinese fir (*Cunninghamia lanceolata*) stands; data represent means ± SE (*n* = 5).

**Site**		**Sapling**	**Juvenile**	**Mature**	**Overmature**
Topography	Orientation	East	Southwest	Southeast	South
	Slope angle	25°	20°	24°	22°
	Altitude (m)	21.8	23.6	22.5	20.4
Growth	Average DBH (cm)	1.40 ± 1.24	6.65 ± 1.29	9.59 ± 2.60	15.27 ± 5.59
	Total height (m)	1.96 ± 0.35	6.49 ± 0.96	9.98 ± 1.41	15.19 ± 3.26
Soil	Total N (g kg^−1^)	0.91 ± 0.21	1.36 ± 0.05	1.41 ± 0.33	1.49 ± 0.27
	Ammonium N (mg kg^−1^)	10.73 ± 2.36	13.49 ± 5.08	16.77 ± 3.25	11.54 ± 2.92
	Total P (g kg^−1^)	0.27 ± 0.04	0.24 ± 0.02	0.35 ± 0.02	0.34 ± 0.01
	Available P (mg kg^−1^)	0.69 ± 0.24	0.75 ± 0.21	0.91 ± 0.19	0.84 ± 0.64
	Total K (g kg^−1^)	8.45 ± 2.65	11.56 ± 1.82	10.70 ± 1.32	6.97 ± 1.35
	Available K (mg kg^−1^)	18.4 ± 3.73	27.99 ± 6.05	29.02 ± 8.14	28.44 ± 8.15
	Soil organic matter (g kg^−1^)	27.18 ± 5.23	24.55 ± 4.66	28.47 ± 5.49	29.01 ± 4.53
	pH	4.3	4.4	4.5	4.4

### Sampling

Four Chinese fir plantations were selected for the study. The stand ages were 5, 15, 25, and 35 years, representing sapling, juvenile, mature, and overmature stands, respectively. The plantations were established with spacing of 2 × 2 m between rows and between trees within a row. In each stand, six trees were randomly chosen on the slopes with the same aspect and all trees were located at the same slope position. New fully developed needles were collected from the tips of three branches located in the middle of sunny crowns using 20-m-high retractable pruning shears (refitted with electric power retractable equipment). The average DBH and total height of trees were recorded (Table 1). The whole sampling process started and finished on June 16, 2019. Needles that were used to identify the phyllosphere bacterial communities were collected with sterilized forceps directly into sterile plastic bags and frozen immediately on dry ice. After being transported to the laboratory, these leaf samples were snap-frozen in liquid N_2_ and then stored at −80°C until DNA was extracted. We did not separate the epiphytic and endophytic components of leaves. Thus, DNA was extracted from microbes both on and inside the leaves. Needles used for transcriptome and metabolome analysis were placed on dry ice immediately after collection to avoid degradation, transported to the laboratory, snap-frozen in liquid N_2_ and stored at −80°C until RNA and metabolites extraction processes were completed.

### 16S rRNA Gene Sequencing

Total bacterial DNA was extracted using the Power Soil DNA Isolation Kit (MO BIO Laboratories, San Diego, CA, USA) in accordance with the manufacturer's protocol. The V3–V4 region of the bacterial 16S rRNA gene was amplified with a primer pair (forward primer, 5′-ACTCCTACGGGAGGCAGCA-3′; reverse primer, 5′-GGACTACHVGGGTWTCTAAT-3′) combined with adapter sequences and barcode sequences. The PCR amplification was performed using the following thermal-cycling protocol: initial denaturation at 95°C for 5 min, followed by 15 cycles at 95°C for 1 min, 50°C for 1 min, and 72°C for 1 min, and a final extension at 72°C for 7 min. The PCR products from the first step of the PCR were purified using VAHTS™ DNA Clean Beads. The second round of PCR was performed using the following thermal-cycling program: initial denaturation at 98°C for 30 s, followed by 10 cycles of 98°C for 10 s, 65°C for 30 s, and 72°C for 30 s, and a final extension at 72°C for 5 min. All final PCR products were quantified using the Quant-iT™ dsDNA HS Reagent and were pooled. High-throughput sequencing analysis of bacterial rRNA genes was performed on the purified, pooled samples using an Illumina HiSeq 2500 platform (2 × 250 paired ends) by the Biomarker Technologies Corporation, Beijing, China.

### RNA Sequencing and Analysis

Total RNA was extracted using TRIzol Reagent (Invitrogen, Carlsbad, CA, USA) following the manufacturer's instructions. Sequencing libraries were generated using the NEBNext® Ultra™ RNA Library Prep Kit for Illumina® (NEB, Ipswich, MA, USA) following the manufacturer's recommendations. Index codes were added to attribute sequences for each sample. The clustering of the index-coded samples was performed on a cBot Cluster Generation System using the TruSeq PE Cluster Kit v3-cBot-HS (Illumia) in accordance with the manufacturer's instructions. After cluster generation, the library preparations were sequenced on an Illumina HiSeq 2000 platform and paired-end reads were generated. Raw data (raw reads) in FASTQ format were first processed using in-house Perl scripts. Transcriptome assembly was accomplished using Trinity software (v2.5.1, Haas et al., 2013) with min_kmer_cov set to 2 by default and all other parameters set to default values. Gene function was annotated based on annotations accessed in the Kyoto Encyclopedia of Genes and Genomes (KEGG) database (https://www.genome.jp/kegg) and Clusters of Orthologous Groups (COG) database (https://www.ncbi.nlm.nih.gov/research/cog-project/). All RNA-seq raw data were deposited to the NCBI Sequence Read Archive (SRA, https://www.ncbi.nlm.nih.gov/sra) accession numbers SRR14812903–SRR14812932 under bio-project number PRJNA737303.

### Metabolomic Analysis

A high-resolution tandem mass spectrometer (Triple TOF 5600+, SCIEX, Framingham, MA, USA) was used to detect metabolites eluted from the column. The quadrupole time-of-flight mass spectrometer was operated in both positive and negative ion modes. The curtain gas was set to 30 psi, ion source gas 1 was set to 60 psi, ion source gas 2 was set to 60 psi, and the interface heater temperature was 650°C. For the positive ion mode, the ionspray voltage floating was set to 5000 V. For the negative ion mode, the ionspray voltage floating was set to −4500 V. The mass spectra were acquired in IDA mode. The time-of-flight mass range was from 60 to 1200 Da. The survey scans were acquired at 150 ms intervals, and as many as 12 product ion scans were collected if they exceeded a threshold of 100 counts s^−1^ with a 1+ charge-state. The total cycle time was fixed to 0.56 s. Four time bins were summed for each scan at a pulse frequency value of 11 kHz through monitoring of the 40 GHz multichannel TDC detector with four-anode/channel detection. Dynamic exclusion was set to 4 s. During acquisition, the mass accuracy was calibrated every 20 samples. To evaluate the stability of the liquid chromatography–mass spectrometry (LC–MS) during the entire acquisition period, a quality-control sample (pool of all samples) was analyzed after every 10 samples.

The acquired MS data pretreatments were performed using XCMS software (Smith et al., 2006), including peak picking, peak grouping, retention time correction, second peak grouping, and annotation of isotopes and adducts. The LC/MS raw data files were converted into mzXML format and processed using XCMS, CAMERA, and the metaX toolbox implemented with R software (https://www.r-project.org/). Each ion was identified by combining the retention time and m/z data. Intensities of each peak were recorded and a three-dimensional matrix containing arbitrarily assigned peak indices (retention time–m/z pairs), sample names (observations), and ion intensity information (variables) was generated.

### Data Analyses

Sequencing reads were spliced using FLASH v1.2.11, quality filtering was performed with Trimmomatic v0.33, and chimeras were eliminated using UCHIME v8.1. The operational taxonomic units (OTUs) were defined using a sequence divergence threshold of 3% (i.e., 97% similarity; Edgar, 2010). The representative OTUs were assigned taxonomically using the RDP classifier v.2.2 with the SILVA 16S rRNA gene database (v.115) (Wang et al., 2007; Quast et al., 2012). Venn diagrams, rank abundance curves, and rarefaction curves were used to analyze differences among stands for high-throughput sequencing data using an online bioinformatic pipeline tool, BMKCloud (www.biocloud.net).

To acquire the best discriminant performance of taxa across stand ages of Chinese fir, a Random Forest model was run using the default parameters of the algorithm in R (R package “randomForest,” ntree = 1,000). The Chao1 index and abundance-based coverage estimator (ACE) index are derivatives of the Shannon diversity index that represent the species richness and evenness of a community, while the Simpson index represents community diversity. These indices were calculated using Mothur v.1.30 (http://www.mothur.org/) (Schloss et al., 2009). The 20 highest ranked bacteria at a genus level that showed significant differences (*p* < 0.05) among three individuals were displayed. The unweighted pair-group method with arithmetic means (UPGMA dendrogram) was used to compare the similarity of the bacterial communities using beta-diversity data and the software QIIME v.1.9.1 (Caporaso et al., 2010). To investigate and visualize the distribution of OTUs and dissimilarity of microbial communities among sites, principal coordinates analysis (PCoA) and analysis of similarities (ANOSIM) were performed using the unweighted UniFrac distance matrix calculated by the R package “vegan,” and the permuted *P*-value was obtained with 10,000 permutations. Galaxy LEfSe (http://huttenhower.sph.harvard.edu/lefse/) was used with the default parameters (minimum linear discriminant analysis (LDA) score (log10) = 4.0 and 30 bootstrap iterations for LDA) to identify bacteria that showed differential abundance patterns among stand ages (Segata et al., 2011). The non-parametric factorial Kruskal-Wallis (KW) sum-rank test accompanied by the unpaired Wilcoxon rank-sum were used to identify the taxa with the greatest differences in abundance (*p*-value). PICRUSt (http://huttenhower.sph.harvard.edu/galaxy/tool_runner?toll_id=PICRUSt_normalize) was used to predict bacterial function.

The online KEGG database and Human Metabolome Database (HMDB) were used to annotate the metabolites by matching the exact molecular mass data (m/z) of samples with those from the databases (https://www.genome.jp/kegg; https://hmdb.ca/). Student's *t*-tests were conducted to assess the significance of differences in metabolite concentrations among the four stands. The *p-*value was adjusted for multiple tests using the Benjamini–Hochberg false discovery rate (FDR). Supervised partial least-squares discrimination analysis was conducted using metaX to discriminate the different variables between groups. The variable importance in the projection (VIP) value was calculated, and metabolites that satisfied the following criteria in any pairwise comparison were considered to be significantly changed: (1) ratio ≥ 2 or ratio ≤ 0.5; (2) *q* value < 0.05; (3) VIP value ≥ 1; and (4) annotation with a secondary metabolite. A SparCC correlation analysis between OTU counts and metabolite relative peak areas was conducted using the M2IA platform (Ni et al., 2020). Principal component analysis (PCA) on the metabolite profiles was constructed and performed using R software.

Fragments per kilobase million (FPMK) were used to calculate the relative expression levels of transcriptome sequences. The differentially expressed genes (DEGs) were analyzed using the DESeq R package (1.10.1). The *p*-values were adjusted using the Benjamini–Hochberg FDR. The DEGs were identified with [fold change] ≥ 1.5 and FDR < 0.05 between each comparison. The unigenes of Chinese fir were annotated using the Mercator web tool (https://www.plabipd.de/portal/mercator-sequence-annotation) and then the DEGs were mapped to metabolic pathways using MapMan software (v3.6.0).

## Results

### Changes in Phyllosphere Bacterial Communities

#### Alpha-Diversity Indices

**Figure 1:**
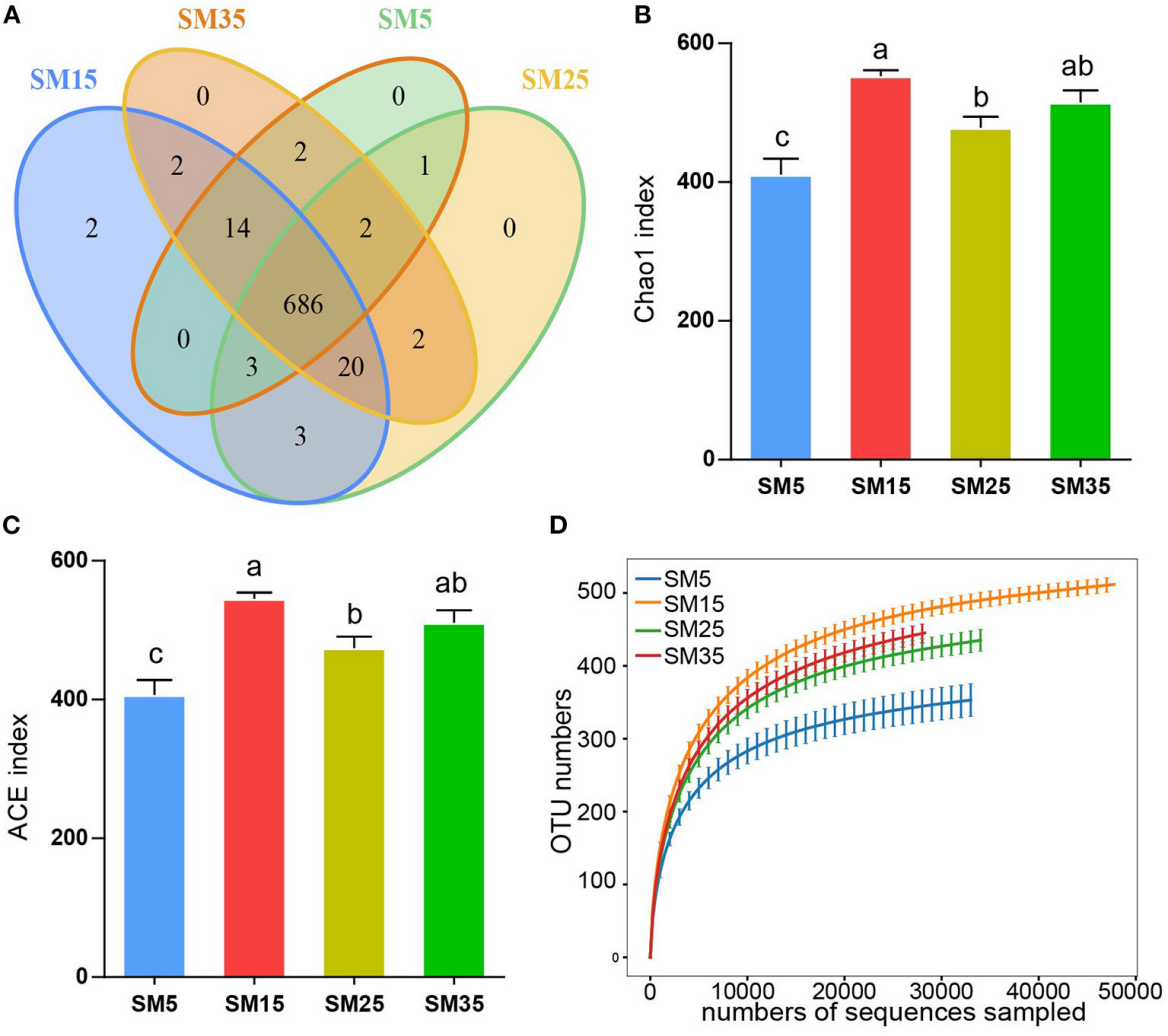
Structure of phyllosphere bacterial communities of Chinese fir in stands of different ages. **(A)** Venn diagram of phyllosphere bacterial operational taxonomic units (OTUs); **(B)** ACE index for each stand age; **(C)** Chaos index for each stand age; **(D)** Rarefaction curve of number of OTUs with increasing sequencing depth. SM5, SM15, SM25, and SM35 represent stands of age 5, 15, 25, and 35 years, respectively (*n* = 18).

Phyllosphere bacterial communities were evaluated by sequencing the 16S rRNA genes in 18 samples from four stands (SM) aged 5, 15, 25, and 35 years (SM5, SM15, SM25, and SM35) (72 samples in total). A total of 737 OTUs were delineated at the 97% similarity threshold. The fewest OTUs (184) were detected in sample SM5-32, and the greatest number of OTUs (572) was detected in sample SM15-61 (Supplementary Figure 1A). Among all OTUs, 686 OTUs were observed in all four stands and two OTUs were specific to the juvenile stand (SM15) (Figure 1A).

The two alpha-diversity indices—ACE and Chao1—showed identical patterns among the stands (Figures 1B,C). For both indices, the alpha diversity of SM5 was significantly lower than that of SM15, SM25, and SM35 (*p* < 0.05); no significant differences were observed between SM15 and SM35 (*p* > 0.05). The two indices for SM25 were significantly lower than those of SM15 (*p* < 0.05) and no significant differences between SM25 and SM35 were detected (*p* > 0.05) (Figures 1B,C). No significant differences in the Shannon and Simpson indices were detected among the four stands (*p* > 0.05), although variation among stands was observed (Supplementary Figures 1B,C).

Rarefaction curves based on the number of OTUs in the bacterial communities attained a saturation plateau, indicating that the sequencing depth was sufficient to represent the majority of microbe species. Species richness was lowest in the SM5 stand and highest in the SM15 stand (Figure 1D). The Shannon index showed a similar pattern with increasing sequencing depth (Supplementary Figure 1D).

#### Beta-Diversity Indices

**Figure 2:**
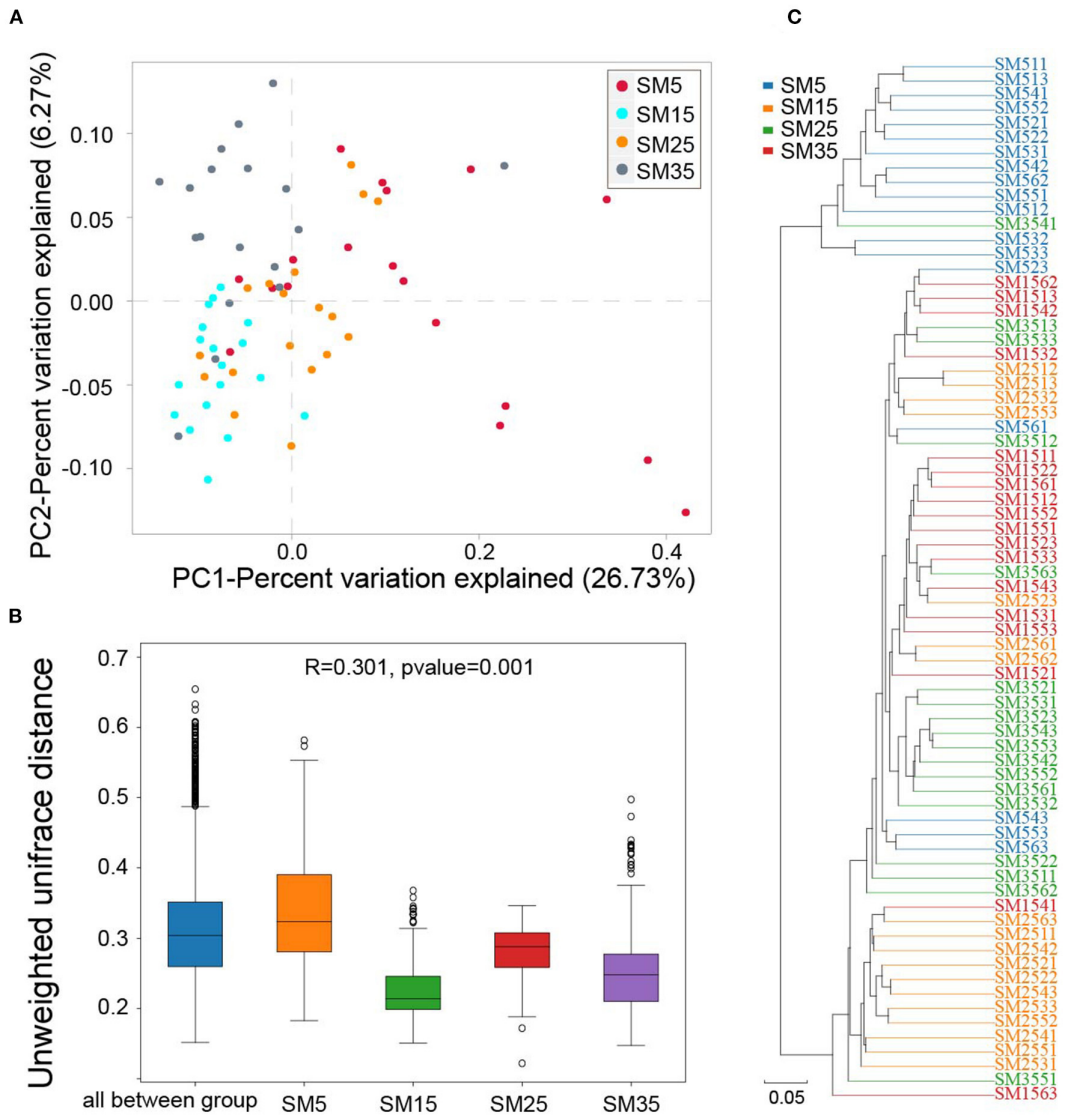
Beta diversity in the phyllosphere bacterial community of Chinese fir. **(A)** Scatterplot of axes 1 and 2 from a PCoA based on unweighted UniFrac dissimilarity matrix; **(B)** unweighted ANOSIM; **(C)** UPGMA dendrogram. SM5, SM15, SM25, and SM35 represent stand ages of 5, 15, 25, and 35 years, respectively.

Figure 2A shows the PCoA of variation in bacterial composition based on the unweighted UniFrac distance matrix. Coordinate 1, representing 26.73% of the variation, was associated with the different stand ages. ANOSIM analysis (*R* = 0.301, *p* < 0.001), also performed using the unweighted UniFrac distance matrix, highlighted significant differences between stand ages (Figure 2B). The results of hierarchical clustering using UPGMA indicated there were distinct differences in the composition of the bacterial communities in the four stands (Figure 2C).

#### Bacterial Distribution at Different Taxonomic Levels and Stand Ages

The predominant phyla comprised Proteobacteria, Cyanobacteria, Bacteroidetes, Actinobacteria, Firmicutes, Verrucomicrobia, Acidobacteria, Armatimonadetes, Patescibacteria, and Deferribacteres, which together accounted for 99.27, 99.64, 99.61, and 99.29% of the bacterial diversity in SM5, SM15, SM25, and SM35, respectively (Supplementary Figure 2A and Supplementary Table 1). The main classes detected comprised Alphaproteobacteria, Oxyphotobacteria, Gammaproteobacteria, Bacteroidia, Actinobacteria, Verrucomicrobiae, Acidobacteriia, Erysipelotrichia, Deltaproteobacteria, and Clostridia, which accounted for 96.89, 98.00, 97.97, and 96.65% of the bacterial diversity in SM5, SM15, SM25, and SM35, respectively (Supplementary Figure 2B and Supplementary Table 1).

The 10 orders that were most abundant comprised Rhizobiales, Chloroflexales, Sphingomonadales, Enterobacteriales, Bacteroidales, Betaproteobacteriales, Pseudomonadales, Verrucomicrobiales, Erysipelotrichales, and Acidobacteriales, which collectively accounted for 78.93, 78.88, 86.18, and 79.22% of the total diversity in SM5, SM15, SM25, and SM35, respectively (Supplementary Figure 2C and Supplementary Table 1).

**Figure 3:**
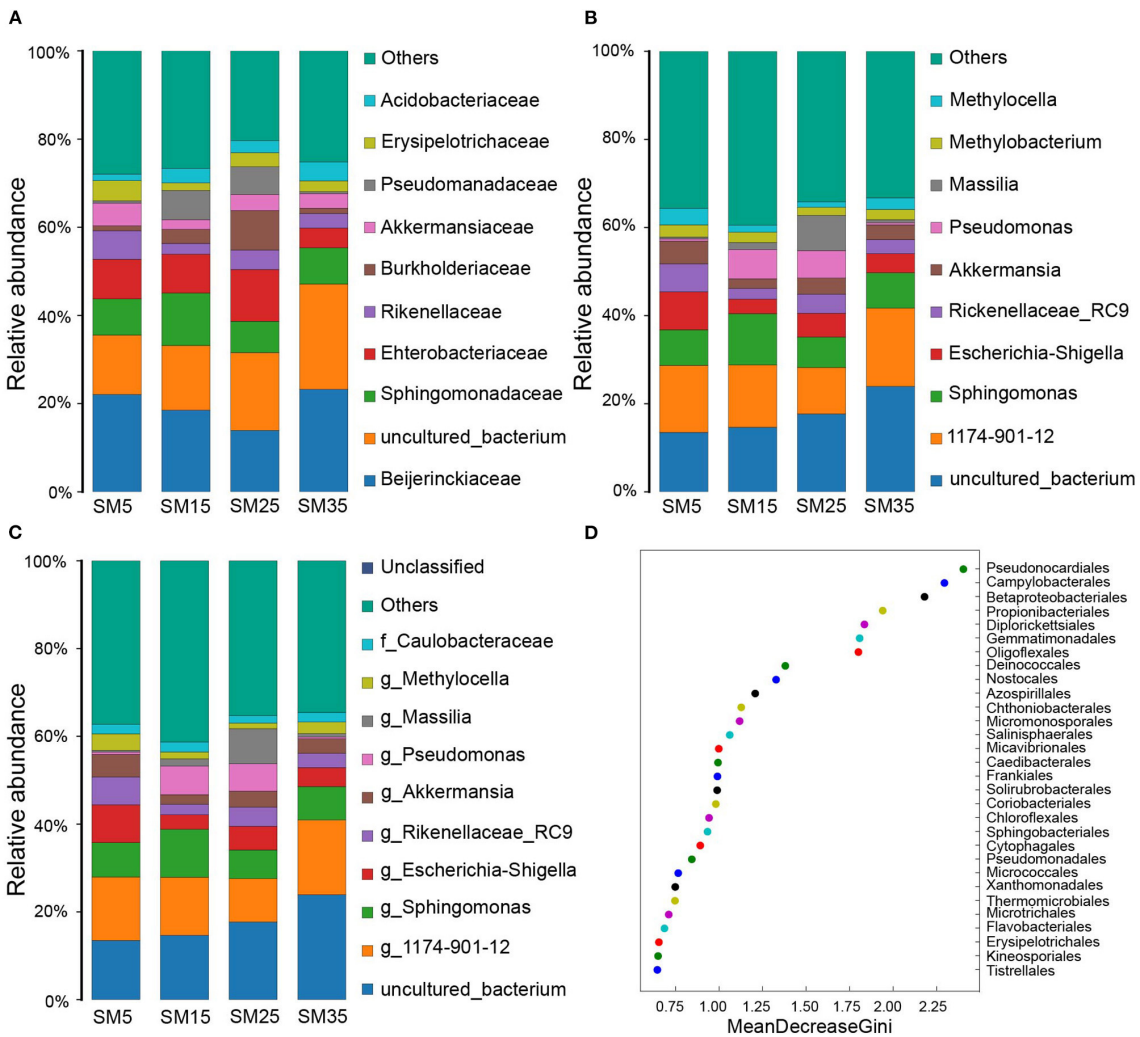
Abundance of phyllosphere bacterial communities with relative abundance > 0.8% as classified to **(A)** family; **(B)** genus; and **(C)** species taxonomic levels. **(D)** The top 30 bacterial orders identified by the predictive Random Forest model using the mean decreasing Gini score. SM5, SM15, SM25, and SM35 represent stand ages of 5, 15, 25, and 35 years, respectively.

The predominant families identified in the phyllosphere bacterial community comprised Beijerinckiaceae, Sphingomonadaceae, Enterobacteriaceae, Rikenellaceae, Burkholderiaceae, Akkermansiaceae, Pseudomonadaceae, Erysipelotrichaceae, and Acidobacteriaceae, which collectively accounted for 58.59, 58.67, 61.94, and 50.90% of the bacterial diversity in SM5, SM15, SM25, and SM35, respectively (Figure 3A and Supplementary Table 1). Of these families, Beijerinckiaceae accounted for the highest percentage abundance (22.05, 18.51, 13.89, and 23.21%, respectively), in the four stands (Figure 3A and Supplementary Table 1).

The predominant identifiable genera comprised *Sphingomonas, Escherichia*–*Shigella*, Rikenellaceae_RC9_gut_group, *Akkermansia, Pseudomonas, Massilia, Methylobacterium*, and *Methylocella*, which together accounted for 35.65, 31.66, 37.62, and 25.03% of the bacterial diversity in SM5, SM15, SM25, and SM35, respectively (Figure 3B and Supplementary Table 1). Of these genera, *Sphingomonas* accounted for the highest percentage abundance (8.07, 11.61, 6.92, and 8.02%, respectively), in the four stands (Figure 3B and Supplementary Table 1).

No bacterial species were identified precisely, but seven species were identified to genus or family level: *Sphingomonas* sp., *Escherichia*–*Shigella* sp., *Akkermansia* sp., *Pseudomonas* sp., *Massilia* sp., *Methylocella* sp., and Caulobacteraceae sp., which collectively accounted for 28.43, 28.39, 32.82, and 21.35% of the total species abundance in SM5, SM15, SM25, and SM35, respectively (Figure 3C and Supplementary Table 1). *Escherichia*–*Shigella* sp. and *Massilia* sp. accounted for the highest percentage in SM5 and SM25 (8.62 and 7.98%), respectively, whereas *Sphingomonas* sp. accounted for the highest percentages in SM15 and SM35 (10.87 and 7.65%, respectively) (Figure 3C and Supplementary Table 1).

A Random Forest model was used to regress the relative abundances of bacterial taxa at the order level against the stand age of Chinese fir. Thirty orders contributed to the four growth stages, including Pseudonocardiales, Campylobacterales, Betaproteobacteriales, Propionibacteriales, Diplorickettsiales, Gemmatimonadales, Deinococcales, and Oligoflexales. They were ranked in decreasing order of importance using the mean decrease Gini score (Figure 3D).

#### LEfSe Analysis of Bacterial Relative Abundances

**Figure 4:**
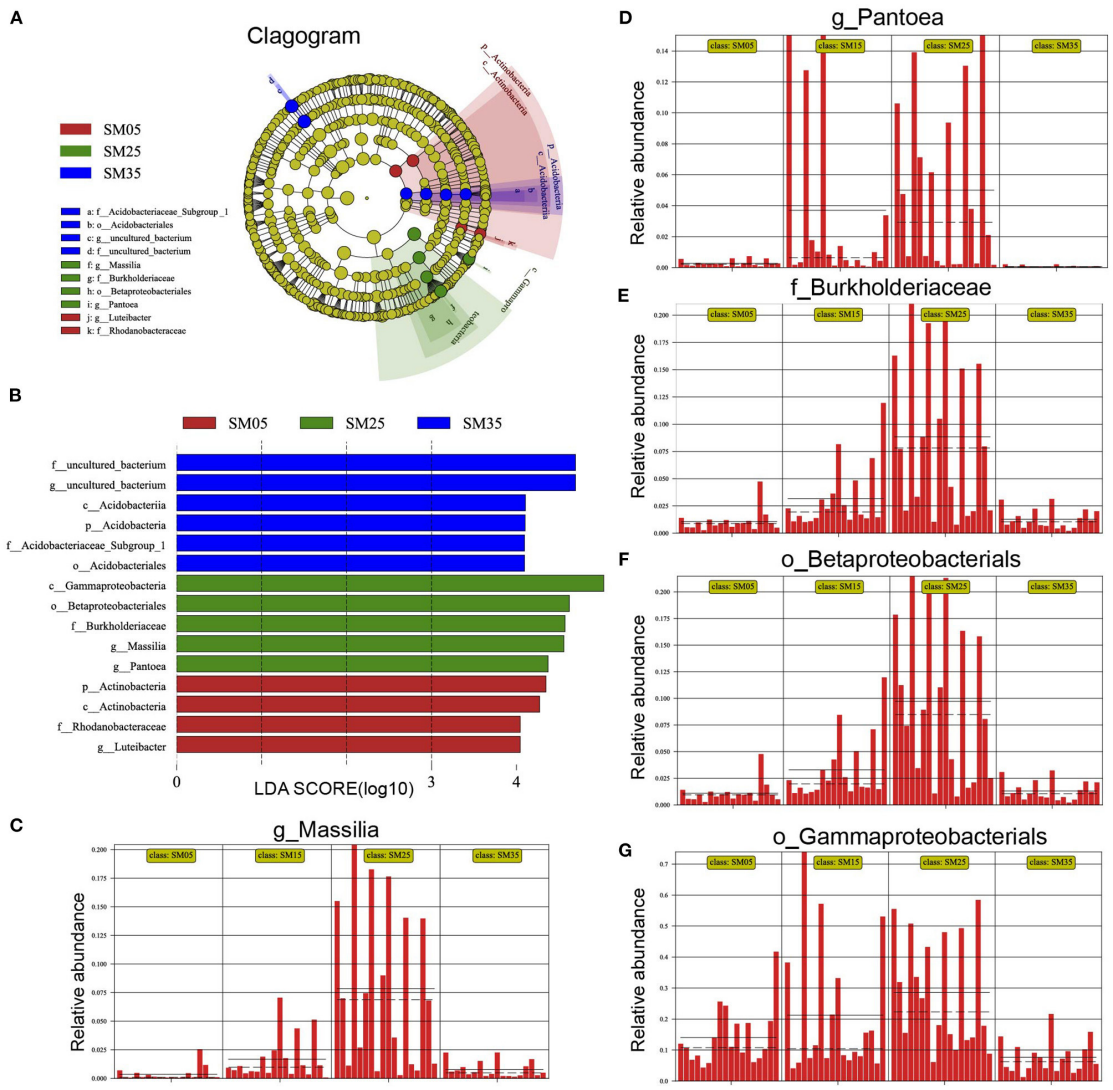
The linear discriminant analysis (LDA) effect size (LEfSe) analysis of bacterial taxa showing significant differences in relative abundance among Chinese fir stands of different ages. **(A)** Cladogram using LEfSe method indicating the phylogenetic distribution of phyllosphere bacterial communities among different ages; **(B)** LDA scores showed the significant bacterial difference among four ages; **(C–G)** the relative abundance of Massilia, Pantoea, Burkholderiaceae, Betaproteobacteriales, and Gammaproteobacteria in each sample from the four groups. SM5, SM15, SM25, and SM35 represent stand ages of 5, 15, 25, and 35 years, respectively.

Changes in stand age induce changes in phytochemical components and environmental factors, resulting in alteration of the phyllosphere bacterial community; therefore, certain bacteria occurred in stands of specific ages. A LDA of effect size (LEfSe) analysis was conducted to detect the bacterial taxa that showed significant differences in relative abundance (Figures 4A,B and Supplementary Figure 4). Members of the genus *Massilia* were rare in sapling stands (SM5) but showed higher relative abundance in mature stands (SM25) (*p* < 0.001) (Figure 4C). *Pantoea* species were rare in overmature stands (SM35) and showed the highest relative abundance in SM25 (*p* < 0.001) (Figure 4D). Members of the family Burkholderiaceae (*p* < 0.001), the order Betaproteobacteriales (*p* < 0.001) and the class Gammaproteobacteria (*p* < 0.001) showed the lowest relative abundances in SM5 and SM35, and the highest relative abundances in SM25 (Figures 4E–G). Species of the family Rhodanobacteraceae (*p* < 0.001) and the genus *Luteibacter* (*p* < 0.001) were detected in the younger stands (SM5 and SM15) but were barely detected in the older stands (SM25 and SM35) (Supplementary Figures 3A,B). Members of the phylum Actinobacteria were detected in all four stands but showed lower relative abundance in SM25 (*p* < 0.001) (Supplementary Figure 3C). Some members of the phylum Acidobacteria, which were also detected in all four stands, showed lower relative abundance in SM5 (*p* < 0.001) (Supplementary Figure 3D).

#### Predicted Functions of Bacterial Communities

**Figure 5:**
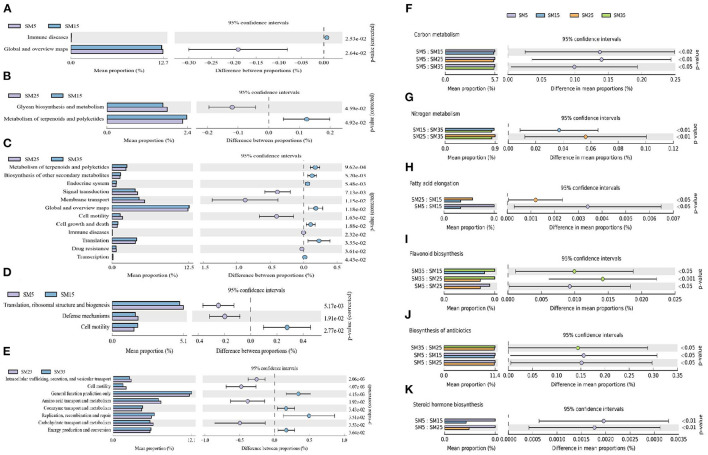
Changes in bacterial functional genes predicted by PICRUSt based on KEGG pathways **(A–C,F–K)** and Clusters of Orthologous Groups (COG) analysis **(D,E)** that differed significantly in pairwise comparisons of Chinese fir stands of different ages. **(F–K)** involved in **(F)** carbon metabolism; **(G)** nitrogen metabolism; **(H)** fatty acid elongation; **(I)** flavonoids biosynthesis; **(J)** antibiotic biosynthesis; and **(K)** steroid hormone biosynthesis, based on third-tier KEGG pathways. SM5, SM15, SM25, and SM35 represent stand ages of 5, 15, 25, and 35 years, respectively.

The software PICRUSt was used to predict variation in functional genes in the phyllosphere bacterial communities. The bacterial communities involved in the metabolic pathways for immune diseases, and global and overview maps showed a significant difference between SM5 and SM15 (*p* < 0.05; Figure 5A). Similarly, bacterial communities showed significant differences in glycan biosynthesis and metabolism, and metabolism of terpenoids and polyketides between SM15 and SM25 (*p* < 0.05; Figure 5B). With an increase in stand age from 25 to 35 years, changes in the phyllosphere bacterial community affected additional metabolic pathways. For 12 metabolic pathways, the bacterial community showed significant differences between SM25 and SM35 (*p* < 0.05; Figure 5C). Significant differences in bacterial communities involved in additional metabolic pathways were detected for other pairwise comparisons of stands (Supplementary Figure 4).

Detailed information on the predicted functional genes in the phyllosphere communities, specifically third-tier KEGG pathways, is shown in Figures 5F–K and Supplementary Figures 4, 5. The carbon metabolism function in SM5 was significantly higher than that in the other three stands (*p* < 0.05; Figure 5F), suggesting that the phyllosphere bacterial community was strongly involved in carbon fixation and transformation in Chinese fir saplings. The nitrogen metabolism function in SM15 and SM25 was significantly higher than that in SM35 (*p* < 0.01; Figure 5G), indicating that the phyllosphere bacterial community was strongly involved in nitrogen absorption and transformation in juvenile and mature stands of Chinese fir. The fatty acid elongation function in SM5 and SM25 was significantly higher than that in SM15 (*p* < 0.05; Figure 5H), suggesting that the phyllosphere bacterial community was less involved in fatty acid biosynthesis in juvenile stands of Chinese fir. The flavonoids biosynthesis function in SM5 and SM35 was significantly higher than that in SM15 and SM25 (*p* < 0.05; Figure 5I), showing that the phyllosphere bacterial community was less strongly involved in flavonoids biosynthesis in the juvenile and mature stands. Similarly, the antibiotics biosynthesis function was significantly higher in SM5 and SM35 than that in the SM15 and SM25 (*p* < 0.05; Figure 5J), indicating that the phyllosphere bacterial community was more strongly involved in antibiotics biosynthesis in sapling and overmature stands. The steroid hormone function in SM5 was higher than that in SM15 and SM25 (*p* < 0.01; Figure 5K), suggesting that the phyllosphere bacterial community was strongly involved in steroid hormone biosynthesis and promoted the functions of steroid hormones in sapling stands of Chinese fir.

Three main comparisons of changes in the predicted functions of proteins of phyllosphere bacterial communities were conducted based on a COG analysis (Figures 5D,E). Three functions (translation; ribosomal structure and biogenesis; defense mechanism; and cell motility) differed significantly between the bacterial communities of SM5 and SM15 (*p* < 0.05; Figure 5D). Between SM15 and SM25, the bacterial communities were involved in 24 functions that showed no significant differences, especially the transport and metabolism of carbohydrates, lipids, inorganic ions, and amino acids (*p* > 0.05). This result suggested that the transition from juvenile to mature stands did not result in significant changes in the bacterial communities involved in these protein functions. However, the transition from mature to overmature stands did result in significant differences in the bacterial communities involved in protein functions associated with transport and metabolism of amino acids and carbohydrates (*p* < 0.05; Figure 5E). The three other pairwise comparisons of stands are summarized in Supplementary Figure 5.

### Foliar Metabolic Profiles in the Four Stands

#### Identification of Metabolites at the Secondary Level

**Figure 6:**
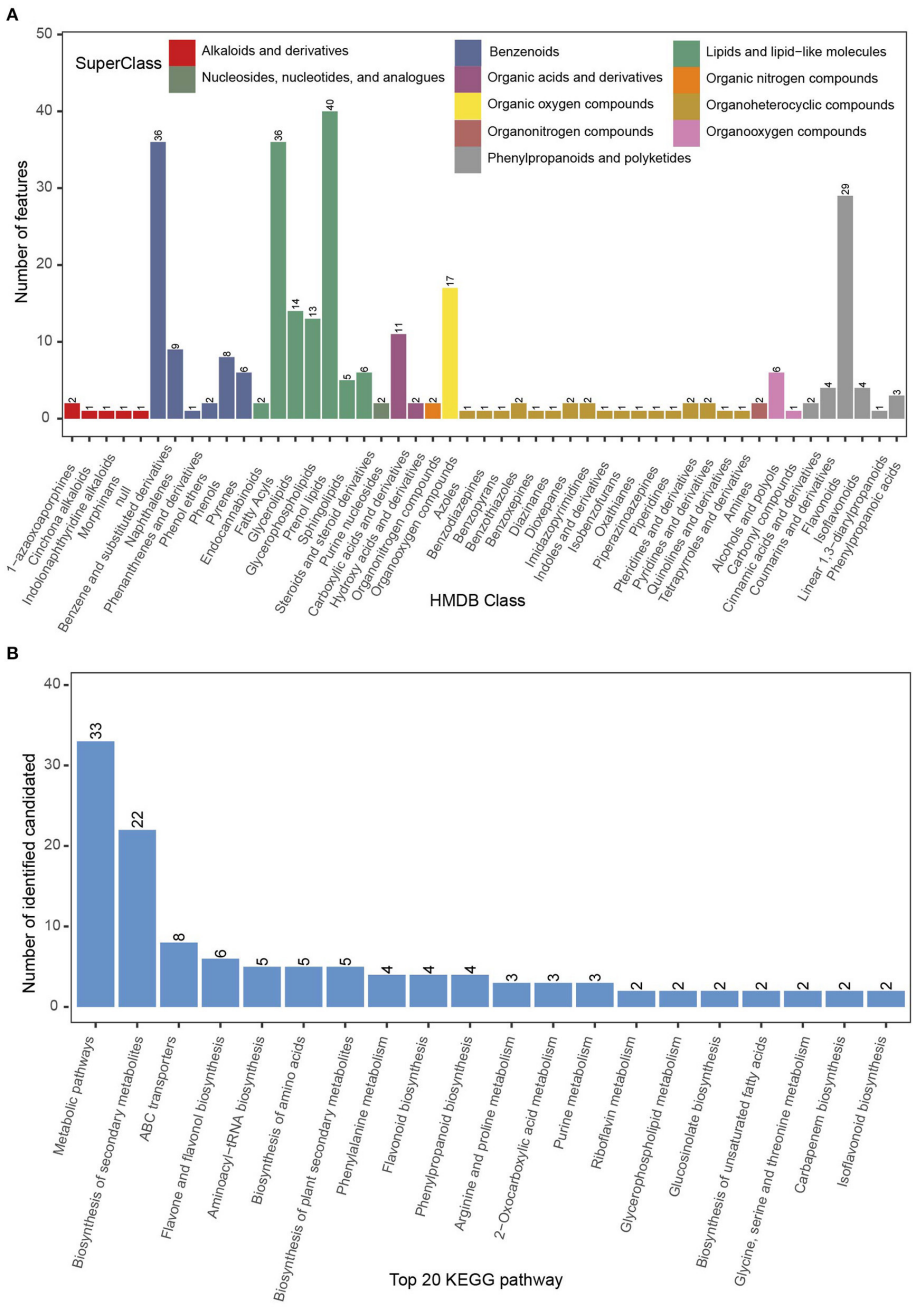
Leaf secondary metabolite profile of the phyllosphere of Chinese fir. **(A)** Metabolite classification based on HMDB data; **(B)** number of candidate metabolites among the 20 highest ranked KEGG pathways.

Phyllosphere metabolites were analyzed by LC/MS (*n* = 36). A total of 292 metabolites were identified from secondary MS: six alkaloids and derivatives (comprising two 1-azaoxoaporphines, one cinchona alkaloid, one indolonaphthyridine alkaloid, one morphinan, and one null), 62 benzenoids (36 benzene and substituted derivatives, nine naphthalenes, one phenanthrene and derivative, two phenol ethers, eight phenols, and six pyrenes), 116 lipids and lipid-like molecules (comprising two endocannabinoids, 36 fatty acyls, 14 glycerolipids, 13 glycerophospholipids, 40 prenol lipids, five sphingolipids, and six steroids and steroid derivatives), two nucleosides, nucleotides, and analogs (two purine nucleosides), 13 organic acids and derivatives (comprising 11 carboxylic acids and derivatives, and two hydroxyl acids and derivatives), two organic nitrogen compounds, 17 organic oxygen compounds, 22 organoheterocyclic compounds (comprising one azole, one benzodiazepine, one benzopyran, two benzothiazoles, one benzoxepine, one diazinane, two dioxepanes, two imidazopyrimidines, one indole, one isobenzofuran, one oxathiane, one piperazinoazepine, one piperidine, two pteridines and derivatives, two pyridines and derivatives, one quinoline or derivative, and one tetrapyrrole or derivative), two organonitrogen compounds (two amines), seven organooxygen compounds (six alcohols and polyols, and one carbonyl compound), and 43 phenylpropanoids and polyketides (comprising two cinnamic acids and derivatives, four coumarins and derivatives, 26 flavonoids, four isoflavonoids, one linear 1,3-diarylpropanoid, and three phenylpropanoic acids) (Figure 6A).

In the KEGG database, 33 metabolites were matched to metabolic pathways, 22 metabolites to the biosynthesis of secondary metabolites, eight metabolites to ABC transporters, six metabolites to flavone and flavonol biosynthesis, and five metabolites to the three pathways: aminoacyl-tRNA biosynthesis, biosynthesis of amino acids, and biosynthesis of plant secondary metabolites (Figure 6B).

#### Comparison of Metabolite Profiles Among the Four Stands

**Figure 7:**
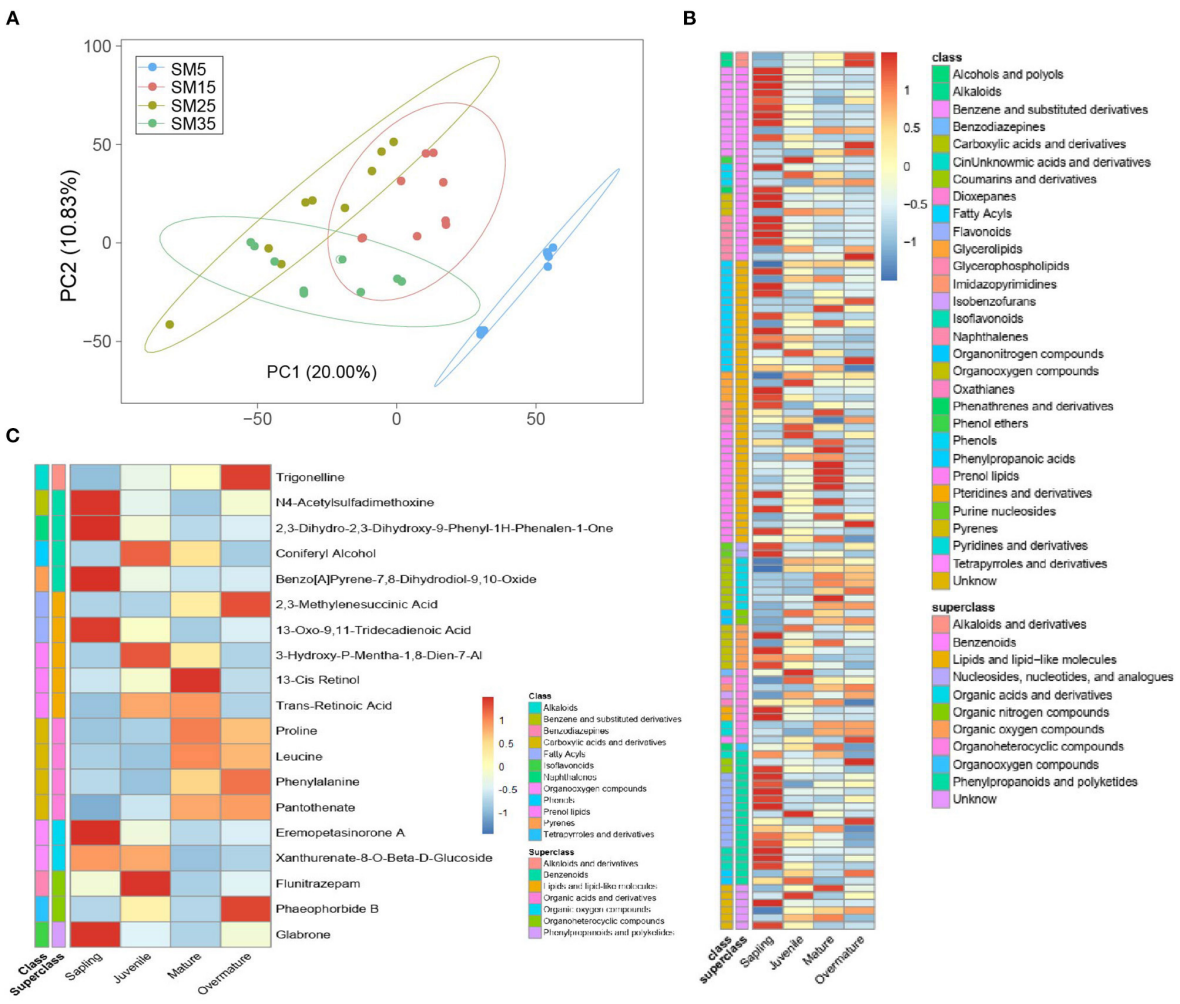
Metabolites that showed a significant change between any two stands of Chinese fir as indicated by partial least-squares discriminant analysis. **(A)** Principal component analysis (PCA) of the foliar metabolites of 28 independent samples collected from Chinese fir aged 5, 15, 25, and 35 years; **(B)** heatmap for all significantly changed metabolites. Metabolites satisfied the following conditions in pairwise comparisons: (1) ratio ≥ 2 or ratio ≤ 0.5; (2) *q* value < 0.05; (3) VIP ≥ 1; (4) annotation with a secondary metabolite; **(C)** level of metabolites that significantly changed at least four times in six pairwise comparisons.

A principal component analysis over the metabolome profiles of the 27 samples analyzed was performed. The first and second principal components explain 20.0% and 10.83% of the variance among samples, respectively (Figure 7A). Partial least-squares discriminant analysis was used to discriminate differences in metabolites between two stands. In total, 112 metabolites showed significant differences between stands. These metabolites belonged to 10 superclasses, namely phenylpropanoids and polyketides, organooxygen compounds, organoheterocyclic compounds, organic oxygen compounds, organic nitrogen compounds, organic acids and derivatives, lipid, and lipid-like molecules, benzenoids, alkaloids and derivatives, and nucleosides, nucleotides, and analogs (Supplementary Figure 6 and Supplementary Table 2). Most benzenoids, dioxepanes, flavonoids, and phenols showed higher concentrations in the sapling stand (SM5) than those in the three other stands (Figure 7B).

Nineteen metabolites that were identified at least four times in six pairs of +comparisons were marked. These metabolites included one alkaloid (trigonelline), four benzenoids (2,3-dihydro-2,3-dihydroxy-9-phenyl-1*H*-phenalen-1-one, coniferyl alcohol, benzo[a]pyrene-7,8-dihydrodiol-9,10-oxide, and N4-acetylsulfadimethoxine), five lipids and lipid-like molecules (2,3-methylene succinic acid, 3-hydroxy-*p*-mentha-1,8-dien-7-al, 13-oxo-9,11-tridecadienoic acid, 13-*cis* retinol, and *trans*-retinoic acid), four organic acids and derivatives (proline, leucine, phenylalanine, and pantothenate), two organic oxygen compounds (eremopetasinorone A and pheophorbide B), and one polyketide (glabrone) (Figure 7C). Among these metabolites, the concentrations of trigonelline, proline, leucine, and phenylalanine increased with stand age.

### Relationship Between Phyllosphere Bacterial Communities and Foliar Metabolites

**Figure 8:**
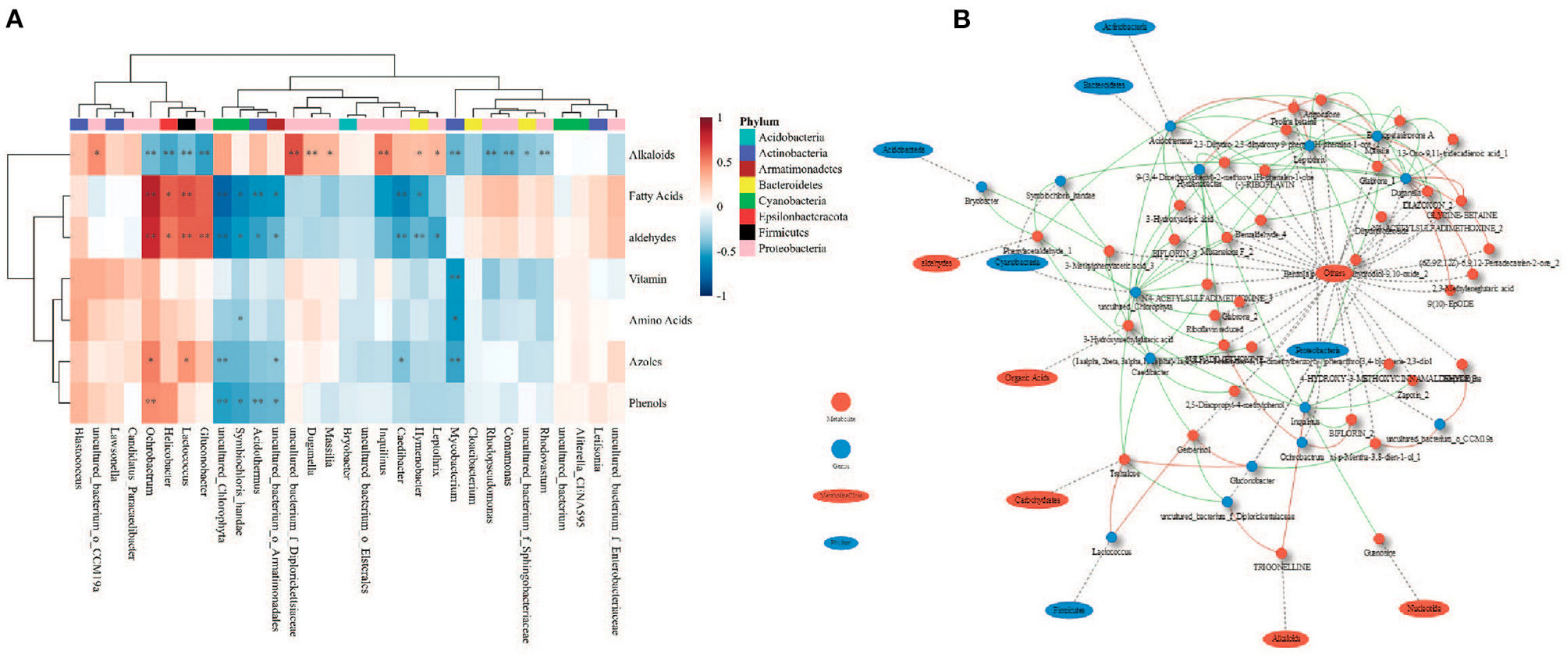
Relationship between metabolites and phyllosphere bacterial communities of Chinese fir. **(A)** SparCC correlations between metabolite classes and genera; **(B)** network constructed for strongly correlated genera and metabolites (|*R*| > 0.6). *p < 0.05, **p < 0.01. Red lines indicate positive relationships; green lines indicate negative relationships.

Phyllosphere bacterial communities were strongly correlated with foliar metabolites (Figure 8). Fatty acids were positively correlated with the genera *Ochrobactrum* and *Lactococcus* (*R* > 0.6), but negatively correlated with an uncultured genus belonging to the family Chlorophyta and the genus *Caedibacter* (*R* < −0.6) (Figure 8A). Alkaloids were positively correlated with an uncultured genus belonging to the family Diplorickettsiaceae (*R* > 0.6) (Figure 8A). Aldehydes were positively correlated with the genus *Ochrobactrum* (*R* > 0.6; Figure 8A). Interestingly, few genera were correlated with certain metabolites, either positively or negatively. A genus uncultured_Chlorophyta belonging to the phylum Cyanobacteria was negatively correlated with 22 metabolites (*R* < −0.6) but was positively correlated with alkaloids (*R* = 0.615) (Figure 8B). The genus *Duganella* was positively correlated with eight metabolites (*R* > 0.6) but was negatively correlated with 13 metabolites (*R* < −0.6). The genus *Hymenobacter* was negatively correlated with 17 metabolites (*R* < −0.6) but was positively correlated with proline betaine (*R* = 0.639). The genus *Massilia* was positively correlated with three metabolites (*R* > 0.6) but was negatively correlated with 15 metabolites (*R* < −0.6).

### Transcriptome Analysis of Chinee Fir Leaves in Different Stand Ages

#### Sequencing, Assembly, and Unigene Functional Annotation

A total of 143.39 Gb clean data were obtained and selected for further analysis after low quality reads were filtered out. Finally, 100,049 unigenes with a mean length of 810 bp were assembled: 42,485 (42.36%) unigene lengths ranged from 200 to 500 bp, 32,030 (32.01%) unigene lengths ranged from 500 to 1,000 bp, and 26,634 (25.63%) unigenes exceeded 1,000 bp. Among the 100,049 unigenes, 83,741 (83.70%) genes were annotated in public databases, including 83.05, 72.77, 60.17, 48.47, 37.11, 28.05, 27.04, and 24.37% in NCBI non-redundant protein sequences (nr), evolutionary genealogy of genes: Non-supervised Orthologous Groups (eggNOG), gene ontology (GO), Pfam, clusters of euKaryotic Orthologous Groups (KOG), a manually annotated and reviewed protein sequence database (SwissProt), COG, and KEGG (Table 2).

**Table 2:** Summary statistics of annotations for Chinese fir.

**Database**	**Number annotated**	**Annotated unigene ratio(%)**
COG	27,050	27.04%
GO	60,198	60.17%
KEGG	24,378	24.37%
KOG	37,128	37.11%
Pfam	48,497	48.47%
Swissprot	28,064	28.05%
eggNOG	72,810	72.77%
nr	83,086	83.05%
All	83,737	83.70%

#### Identification and Functional Enrichment of Differentially Expressed Genes

**Figure 9:**
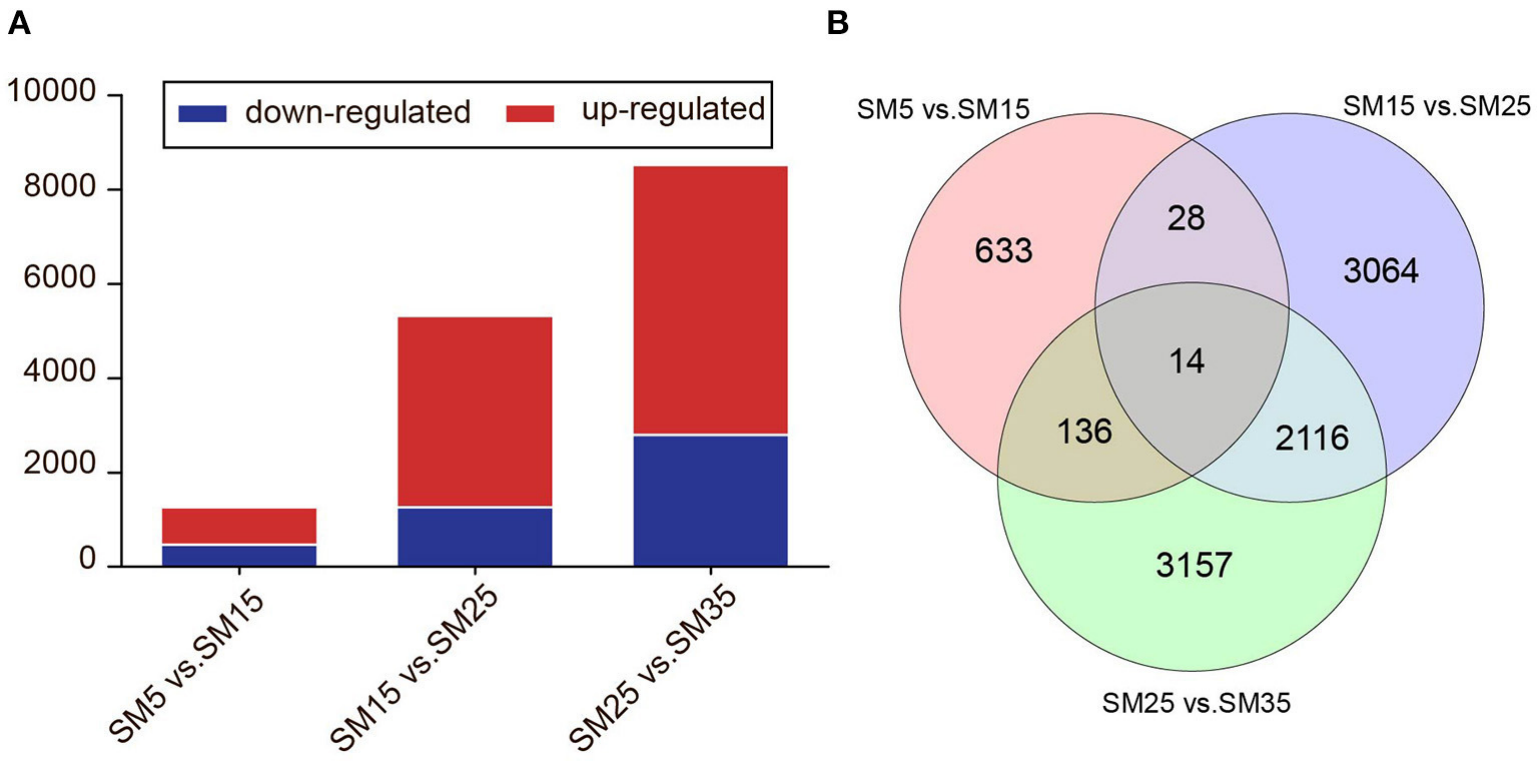
Expression of unigenes in Chinese fir at four stages. **(A)** Numbers of DEGs in each comparison; **(B)** Venn diagram of analyses of differentially and stage-specific expression genes per comparison. SM5, SM15, SM25, and SM35 represent stand ages of 5, 15, 25, and 35 years, respectively.

To identify the Chinese fir genes that were significantly up or down-regulated at different stand ages (SM5, SM15, SM25, and SM35), DEGs were identified with a [fold change] ≥ 1.5 and an FDR < 0.05 between each comparison using DESeq. As shown in the Venn diagram in Figure 9A, we identified 469 downregulated and 792 upregulated DEGs between SM5 and SM15. Similarly, 1,265 downregulated and 4,057 upregulated DEGs and 2,799 downregulated and 5,724 upregulated DEGs were obtained between SM15 and SM25 and between SM25 and SM35, respectively. The highest number of DEGs were identified between SM25 and SM35. A large number of DEGs were stand age-specific. There was 633, 3,064, and 3,157 DEGs for SM5 vs. SM15, SM15 vs. SM 25, and SM25 vs. SM35, respectively (Figure 9B).

**Figure 10:**
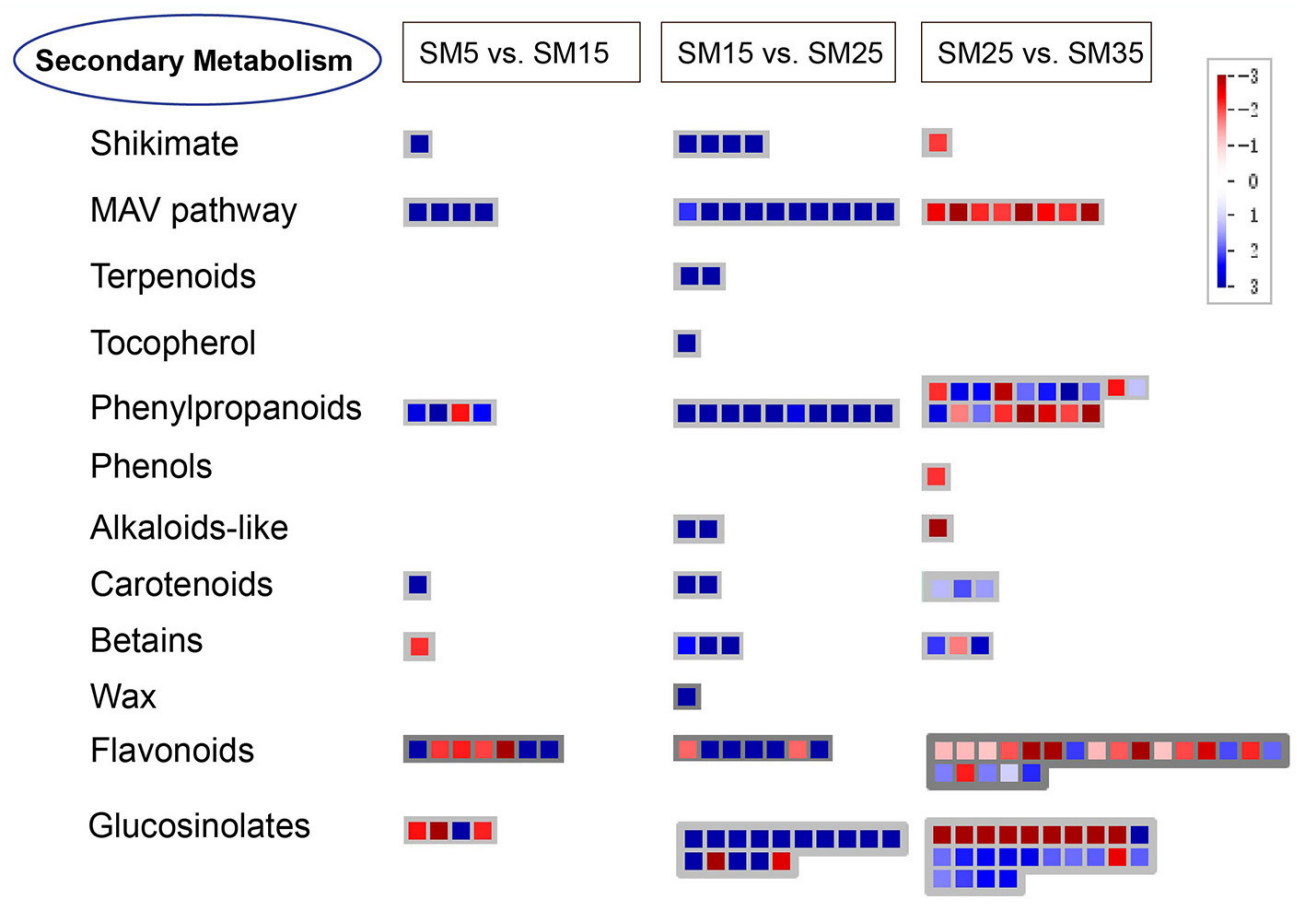
Schematic diagram of the transcriptional changes of genes involved in secondary metabolism in leaves of Chinese fir at different stand ages. The log_2_FC of DEGs in SM15, SM25, and SM35 compared with SM5, SM15, and SM35 is presented using the MapMan visualization platform. SM5, SM15, SM25, and SM35 represent stand ages of 5, 15, 25, and 35 years, respectively.

All DEGs from the three groups (SM5 vs. SM15, SM15 vs. SM25, and SM25 vs. SM35) were assigned to MapMan functional categories. According to the metabolomics results (Figures 7B,C), pathways related to alkaloids, phenylpropanoid, flavonoids and others were analyzed to help understand the secondary metabolism of Chinese fir in different stand ages. Figure 10 presents a schematic view of some of the secondary pathways of DEGs in response to the different stand ages of Chinese fir. Detailed information is listed in Supplementary Table 3.

In the shikimate acid pathway, EMB1144 (at1g51410) was significantly up-regulated in SM15 (vs. SM5); genes like P-loop containing nucleoside triphosphate hydrolases superfamily protein (at2g16790), 3-phosphoshikimate 1-carboxyvinyltransferase activity (at2g45300), and EMB1144 (at1g48850) were significantly activated in SM25 compared with SM15; while the EMB1144 (at1g51410) gene was clearly inhibited in SM35 (vs. SM25). Phenolic compounds are biosynthesized by the shikimate pathway. One gene possessed of laccase activity (at1g18140) was up-regulated in SM35 (vs. SM25) in a simple phenol biosynthesis process.

In the mevalonate (MAV) pathway, genes encoding the thiolase family protein (at5g47720), HMG-CoA synthase protein (at4g11820), GHMP kinase family protein (at3g54250), and a protein with isopentenyl diphosphate:dimethylallyl diphosphate isomerase activity (at3g02780) were significantly up-regulated in SM15 compared with SM5. Ten other genes encoding acetoacetyl-CoA thiolase 2 (at5g48230 and at5g48230), thiolase family protein (at5g47720), a protein with hydroxymethylglutaryl-CoA synthase (at4g11820), and mevalonate kinase (MK) (at5g27450) were significantly up-regulated in SM25 (vs. SM15); but eight genes encoding proteins of hydroxymethylglutaryl-CoA synthase activity (at4g11820, at4g11820, and at4g11820), farnesyl diphosphate synthase 2 (at4g17190), acetoacetyl-CoA thiolase 2 (at5g48230), GHMP kinase family protein (at3g54250), and with isopentenyl diphosphate:dimethylallyl diphosphate isomerase activity (at3g02780) were down-regulated in SM35 compared with SM25. The MAV pathway plays a critical role in synthesizing the precursor of terpenoid. As we can see from Figure 9 and Supplementary Table 3, there were four genes encoding cycloartenol synthase 1 (CAS1) (at2g07050) in the terpenoids pathway, beta-ketoacyl reductase 1 (KCR1) (at1g67730) in the wax pathway and 4-hydroxyphenylpyruvate dioxygenase (4HPPD) (at1g06570) in tocopherol biosynthesis, which were up-regulated exclusively in SM25 (vs SM15). Two genes, pyridoxal phosphate (PLP) (at2g20340) and oxygenase superfamily protein (at3g46490), on the alkaloids-like pathway were clearly up-regulated in SM25 (vs. SM15), and the expression of gene pyridoxal phosphate (PLP) (at2g20340) was significantly down-regulated in SM35 (vs. SM25).

The carotenoid biosynthesis pathway catalyzes the synthesis of essential pigments, which are crucial for light harvesting and photoprotection in photosynthetic organisms. With the growth of Chinese fir, the gene encoding cytochrome P450, family 709, subfamily B, polypeptide 2 (CYP709B2) (at2g46950) was highly activated in SM15 (vs SM5). This over-expression also occurred in SM15 (vs SM5), including cytochrome P450, family 76, subfamily C, polypeptide 1 (CYP76C1) (at2g45560), and cytochrome P450, family 97, subfamily A, polypeptide 3 (CYP97A3) (at1g31800); and SM35 (vs. SM25), including phytoene desaturase 3 (PDS3) (at4g14210), lutein-deficient 2 (LUT2) (at5g57030), and carotenoid cleavage dioxygenase 1 (CCD1) (at3g63520) genes encoding cytochrome P450, family 76, subfamily C, polypeptide. All DEGs in the betains pathway were betaine-aldehyde dehydrogenase (at1g74920), and their expression gradually increased as trees grew older.

Flavonoids are a large class of secondary metabolites and are widely distributed in plants. The growth of the Chinese fir modulated the expression of most structural genes. Two NAD(P)-linked oxidoreductase superfamily proteins (at2g37770, at2g37770) and two cytochrome P450, family 81 proteins (at5g36220, at5g67310) were down-regulated in SM15 (vs. SM5); two flavonoid 3'-hydroxylase (F3'H) belonging to cytochrome P450, family 706, subfamily A (at5g07990) and cytochrome P450, family 76, subfamily C (at2g45550), and one NAD(P)-linked oxidoreductase superfamily protein (at3g53880) were significantly up-regulated in SM15 (vs. SM5). Dihydroflavonol 4-reductase (DFR) (at4g35420), flavonoid 3'-hydroxylase (F3'H) (at2g45560) and three NAD(P)-linked oxidoreductase superfamily proteins (at2g37770, at2g37770, and at2g37770) were up-regulated in SM25 compared with SM15, one dihydroflavonol 4-reductase (DFR) (at4g27250) was obviously down-regulated in SM25 compared with SM15. Four flavonol 3-O-glucosyltransferase (at5g65550, at1g79370, at5g65550, and at5g49690), three NAD(P)-linked oxidoreductase superfamily proteins (at2g37790, at2g37770 and at2g37770), three flavonoid 3'-hydroxylase (F3'H) (at2g45550, at3g48280, and at2g45560), and two dihydroflavonol 4-reductase (DFR) (at5g42800, and at1g51410) were down-regulated in SM35 (vs SM25), while three flavonoid 3'-hydroxylase (F3'H) (at2g23220, at2g45550 and at3g52970), NAD(P)-binding Rossmann-fold superfamily protein (at1g75290), UDP-glycosyltransferase 73B4 (UGT73B4) (at2g15490) and cytochrome P450, family 705, subfamily A (at2g27010) were up-regulated in SM35 compared with SM25.

Phenylalanine ammonia lyase (PAL) is the first enzymatic step of phenylpropanoid biosynthesis that begins with the deamination of phenylalanine to yield cinnamic acid. Compared with SM5, PAL (at5g04230), O-methyltransferase 1 (OMT1) (at5g54160) and cytochrome p450 81d1 (CYP81D1) (at5g36220) were greatly up-regulated in SM15. In SM25, all DEGs were up-regulated compared with SM15, including 4-coumarate–CoA ligase 1(4CL1) (at3g21230 and at1g20500), elicitor-activated gene 3-2 (ELI3-2) (at4g37990 and at4g37980), and cinnamyl alcohol dehydrogenase (at4g37970, at4g34230, at4g34230, and at2g21730). In SM35, more than half of DEGs were down-regulated compared with SM15 (Supplementary Table 3).

## Discussion

### Analysis of Chinese Fir Phyllosphere Bacterial Communities at Four Stand Ages

The phyllosphere provides habit for a variety of microbes that affect nutrient cycling and plant health (Qin et al., 2019; Gong and Xin, 2020; Lajoie et al., 2020). In this study, the relationships between the phyllosphere bacterial community and phyllosphere metabolites of Chinese fir at different stand ages were explored. During stand growth, the phyllosphere bacterial community and metabolic profile differed significantly among the four growth stages. However, most bacteria and metabolites showed non-linear relationships with stand age (Figures 3, 7). This was primarily because competition among individuals in old stands was greater than that in young stands; thus, trees in old stands must translocate greater quantities of nutrients owing to interspecific competition, and their bacterial communities face additional stress from secondary metabolites (Chen and Wang, 2013). The phyllosphere bacterial diversity decreased from the juvenile to the mature stages and increased from the mature to the overmature stages (Figures 1B,C). These trends predominantly reflect that self-thinning begins at the juvenile to mature stages, which increases the secondary metabolites concentration and suppresses bacterial diversity (Sun et al., 2011). The variation in phyllosphere bacterial diversity with stand age observed in the present study is consistent with variation in the soil bacterial diversity of Chinese fir plantations, which indicates that the growth status of Chinese fir may influence microhabitats and, consequently, the microbes that inhabit those microhabitats (Wang C. Q. et al., 2019).

Although the phyllosphere communities at the four growth stages comprised similar bacterial members, distinct differences were observed in alpha and beta diversity, which indicated that the phyllosphere bacterial composition was unique at each stand age (Figure 1) (Delhaes et al., 2012). The primary reason for the shift in the bacterial community composition is nutritional changes: net photosynthesis in conifers decreases with stand age (Greenwood et al., 2008; Räim et al., 2012). Hence, bacterial carbon metabolism was highest at the sapling stage, and the limited leaf area promoted antibiotic biosynthesis at the sapling stage (Figures 5F,J). The nitrogen:phosphorus ratio in the leaf generally increases with stand age (Zhang et al., 2015, 2018; Zhou H. et al., 2016), and a relatively high level of nitrogen nutrition decreases the bacterial nitrogen metabolism function. Most variable metabolites were associated with metabolic and secondary metabolites biosynthesis pathways (Figure 5B). Previous research indicates that the dominant bacteria in the phyllosphere of conifer needles are not only similar across stand ages, but also between locations (Rastogi et al., 2012). This similarity may be caused by the stability of cuticular wax chemicals (e.g., long-chain hydrocarbons), which provide a constant environment for bacteria (Tinto et al., 2017; Wang et al., 2018).

The genera *Sphingomonas, Pseudomonas, Massilia, Methylobacterium, Methylocella*, and *Akkermansia* showed high relative abundances at all stand ages (Figure 3B). This result is similar to those reported by Purahong et al. (2016) and Tláskal et al. (2016). These authors reported that the relative abundances of the genera *Sphingomonas, Pseudomonas*, and *Massilia* were higher in juvenile and mature stands than in sapling and overmature stands.

Members of the genus *Methylobacterium* perform various functions, such as inhibition of pathogenic bacteria (García-Coca et al., 2020), nitrogen fixation (Sy et al., 2001), and pollutant degradation (Lu et al., 2019). However, their functions when they colonize leaves and needles remain unclear. Given that phyllospheric *Methylobacterium* bacteria contain ultraviolet A-absorbing compounds (Yoshida et al., 2017), these bacteria may increase the resistance of leaves and needles to oxidative stress caused by high light intensity. Thus, the higher relative abundance of *Methylobacterium* bacteria in the SM5 stand (Figure 3B) is beneficial for sapling resistance to oxidative stress. Members of the genus *Pseudomonas* are often used as biocontrol agents (De Vrieze et al., 2019; Palyzová et al., 2019; Liang et al., 2020), and these bacteria may influence leaf surface permeability and cuticle development (Xiao et al., 2004; Schreiber et al., 2005). Some members of the genus *Sphingomonas* are facultative phototrophs (Yabuuchi and Kosako, 2015) and are capable of suppressing infection by pathogenic bacteria (Innerebner et al., 2011). The aforementioned results suggest that the phyllosphere bacterial community may provide a defensive barrier to protect Chinese fir from pathogens and oxidative stress caused by high light intensity.

### Metabolic Profile of Chinese Fir in Different Growth Stages

Chinese fir trees grow rapidly from the sapling to the juvenile stages. Fifty metabolites differed between the sapling and juvenile stands, whereas 26 metabolites differed between each stand age after the juvenile stage (Supplementary Figure 6). These results suggest that the change in nutrient requirements and the nutrient distribution strategy may be reflected in the metabolite profiles (Zhou et al., 2016a). This age-related variation has been documented in Anatolian black pine (*Pinus nigra* subsp *pallasiana*) and Norway spruce [*Picea abiess* (L.) Karst.] (Köstner et al., 2002; Turfan et al., 2018). The weakening of the strong relationship between stand age and many metabolites at the overmature stage suggested that metabolic activities changed after the tree attained maturity, although nutrients such as nitrogen and phosphorus continue to accumulate in newly developed needles (Zhou et al., 2016b).

Quantities of benzenoids, including benzaldehyde, phenylacetaldehyde, and salicylic acid, were significantly higher in the sapling stand (Supplementary Table 2). These compounds show antimicrobial activity, which suggests that saplings biosynthesize a greater number of defense molecules in response to bacterial colonization (Whipps et al., 2008; Chaturvedi et al., 2012). Phenylalanine is the precursor of lignin and acts as a channel for carbon sequestered by photosynthesis (Pascual et al., 2016). The higher phenylalanine concentration in mature and overmature stands indicated that the needles contained a higher content of lignin (Figure 7C). The content of trigonelline also increased with stand age. Trigonelline may act as a nutrient source, cell-cycle regulator, compatible solute, a bioactive substance for nyctinastic leaf movement, and a signal transducer, and may play a role in detoxification of nicotinate and nicotinamide (Ashihara et al., 2015). Trigonelline accumulates during leaf and fruit maturation in *Coffea arabica* Linn. (Zheng et al., 2004). Therefore, trigonelline or its correlated OTUs (e.g., the genera *Pantoea, Cedecea SS01, Massilia*, and *Pseudomonas*) may be useful to estimate the degree of tree development in Chinese fir.

Flavonoids, which function as antioxidants, may protect leaves from ultraviolet irradiation damage. Such protection is important for saplings because the individual leaves of saplings may be exposed to higher incident light intensity than the leaves of older trees (Ma et al., 2014). We observed that bacterial flavonoids synthesis activity was high in the sapling and overmature stands (Figure 5I), and the concentrations of most flavonoids were high in the sapling stand (Figure 7B). However, flavonoids biosynthesis in the leaves showed little variation during growth at the transcript level; only three genes encoding enzymes associated with flavonoids biosynthesis were highly expressed at the juvenile and mature stages (Supplementary Figure 7), which was inconsistent with flavonoid concentrations determined at the four growth stages. These results suggest that phyllosphere bacteria produce and furnish leaves with a plethora of flavonoids in the early growth stage of Chinese fir. Consequently, saplings may have less need to synthesize enzymes involved in flavonoids synthesis and can allocate a greater proportion of nutrients to primary metabolism for growth.

### Regulatory Relationship Between Microbial Communities and Their Host

Numerous studies have focused on the roles of endophytes in roots, stems and leaves, or rhizosphere microorganisms, to ascertain their influence on plant secondary metabolites (PSMs). For example, the *Echinacea purpurea* microbiome (bacterial strains isolated from stems and leaves) interaction model showed that the microbiome affected the production of volatile organic compounds, phenylpropanoids, and alkamides in the plants (Maggini et al., 2017, 2019a,b). Korenblum et al. (2020) revealed a plant metabolism-related epiphytic leaf microbiota, finding that local colonization of roots by bacteria in the genus *Bacillus* triggered a systemic exudation of acylsugar secondary metabolites in tomatoes. Gargallo-Garriga et al. (2016) analyzed the foliar metabolomes of leaves of *Sambucus nigra* L. plants before treatment and after 1, 7, 15, and 30 days of fumigation with streptomycin, oxytetracycline and chloramphenicol, and found the concentrations of acetyl-CoA, citraconic acid, isoleucine, and several other PSMs (such as terpenes and phenols in the epiphytic extracts) tended to decrease after treatment. A recent study identified the endophytic and epiphytic microbial taxa associated with seeds and indicated that *Salvia miltiorrhiza* Bunge possessed a distinctive seed-associated microbiome, including *Pantoea, Pseudomonas, Sphingomonas*, and Dothideomycetes; this microbiome contains a gene reservoir related to the synthesis of the terpenoid backbone and other compounds, thus providing additional metabolic capabilities to host plants (Chen et al., 2018). However, our understanding of the effects of the microbiome in the phyllosphere, including plant-microbiome interactions, is still limited. In the current study, some genera of bacterial communities in the phyllosphere were strongly correlated with some categories of foliar metabolites, including alkaloids, fatty acids, aldehydes, vitamins, amino acids, azoles, and phenols. The correlations between metabolites and the microbiome on Chinese fir provide new insights into their functions.

### Changes of Gene Expression in Secondary Metabolism Pathway Over Time

As a key branch-point compound, chorismic acid is the end product of the shikimate pathway.Shikimic and chorismic acids are the common precursors for the synthesis of L-Phe, L-Tyr, L-Trp and diverse phenolic compounds (Santos-Sánchez et al., 2019). Consistent inhibition between the EMB1144 (at1g51410) gene and laccase gene (at1g18140) in the overmature stage indicated a potential correlation. Laccase is involved in the process of reducing oxidation and the lignin catabolic process. It has been demonstrated that LACCASE2 (LAC2) acts as a negative regulator of lignin deposition in *Arabidopsis* root vascular tissues during water deficit (Khandal et al., 2020). Compared with the mature age stands, the inhibition of the laccase gene (at1g18140) implied a decrease in lignin accumulation in the overmature stage. This conflicted with the high level of phenols in the overmature stands but was consistent with the lower expression of several genes related to cinnamyl alcohol dehydrogenase (CAD), which is one of the key enzymes that act in the final stage of lignin monomers biosynthesis (Supplementary Tables 2, 3). Cuticular waxes consist of intracuticular waxes that are embedded in cutin and epicuticular waxes that covered the outmost surfaces of the plant. The epicuticular waxes provide a hydrophobic barrier to protect land plants from environmental stresses (Lee and Suh, 2013). Cuticular waxes are composed of a mixture of hydrocarbons, alkanes, aldehydes, primary and secondary alcohols, ketones, and esters, derived from very-long-chain fatty acids (VLCFAs). As the beta-ketoacyl reductase 1 (KCR1) catalyzes the first reduction during the process of VLCFA synthesis, the excessive expression of at1g67730 indicates a higher level of cuticular waxes in the mature stage. Tocopherols are believed to protect chloroplast membranes from photooxidation and help to provide an optimal environment for the photosynthetic mechanism. Tocopherols are only synthesized by photosynthetic organisms and consist of a polar chromanol ring and a 15-carbon lipophilic prenyl chain derived from homogentisic acid (HGA) and phytyl diphosphate (PDP). HGA is formed from p-hydroxyphenyl pyruvate (HPP) by the cytosolic enzyme HPP dioxygenase (HPPDase) (Fryer, 1992; Garcia et al., 1997; Munné-Bosch and Alegre, 2002). Overexpression of HPPDase (at1g06570) indicates the first committed step to synthesis of plastoquinone and tocopherols, promoting the antioxidant properties of plants. Terpenoids are derived from the universal five-carbon building block, isopentenyl diphosphate (IPP) and its allylic isomer dimethylallyl diphosphate (DMAPP). The mevalonic acid (MVA) pathway is primarily a cytosolic independent pathway to produce IPP and DMAPP in plants (Tholl, 2015). The MVA pathway predominantly provides precursors for terpenoid biosynthesis in mitochondria, such as ubiquinones and polyprenols. Cycloartenol synthase 1 (CAS1) (at2g07050) is involved in the pentacyclic triterpenoid biosynthetic process, catalyzing the reaction from epoxysqualene to cycloartenol. Compared with the juvenile age stands, down-regulation of CAS1 in mature age stands may resulted from the inhibition of related genes in the MVA pathway. Alkaloids are generally extracted from many plants, and they have a variety of biological actions. According to their chemical structure, alkaloids can be classified into piperidine alkaloids, isoquinoline alkaloids, indole alkaloids, terpenoids alkaloids, steroidal alkaloids, and quinoline alkaloids (Liu et al., 2019). Genes related to alkaloids biosynthesis (at2g20340, at3g46490) were up-regulated in mature stands, and the at2g20340 gene was down-regulated in overmature stands, compared with juvenile and mature ages, respectively. This was inconsistent with the high concentration of trigonelline and sampangine (Supplementary Table 2). The two genes did not seem to play determining roles in the alkaloids pathway. Carotenoids are natural isoprenoid pigments that provide leaves, fruit, vegetables, and flowers with distinctive yellow, orange, and some reddish colors as well as certain aromas. They are essential components required for photosynthesis, photoprotection and the production of carotenoid-derived phytohormones, including ABA and strigolactone (Cazzonelli, 2011). In contrast to the other biosynthesis processes in secondary metabolism, the increasing expression of DEGs in the carotenoid pathway indicated the constant accumulation of carotenoid with tree growth. As the Chinese fir trees grew, more genes involved in the phenylpropanoid pathway were activated, but this tendency seemed to be reversed after the mature stage. Compared with mature stands, more than half of the DEGs were inhibited in overmature stands (Figure 10). The production of these genes including cinnamyl alcohol dehydrogenase (CAD), O-methyltransferase (OMT), and NAD(P)-binding Rossmann-fold superfamily protein were crucial in the phenylpropanoid pathway (Supplementary Table 3). A similar trend occurred in the flavonoids synthesis pathway (Figure 10). In response to biological and abiotic stress, the phenylpropanoids biosynthesis pathway produced a variety of secondary metabolites, including flavonoids, monolignols, hydroxycinnamates (HCAs), lignins, and lignans, which acted as components of cell walls, protectants against UV radiation, and signaling molecules phytoalexins against herbivores and pathogens (Vogt, 2010; Deng and Lu, 2017). Research into ginkgo leaves found that increasing tree age was more likely to be detrimental to the manufacture of flavonoids (Zou et al., 2019).

Combining the transcriptome and metabolome results on the changes in secondary metabolism at different ages reflected that Chinese fir was able to continually enhance secondary metabolism with age until maturity, but this ability declined at the overmature stage.

## Conclusions

Our study showed that, under a similar environment, the phyllosphere bacterial community structures and metabolic profiles of Chinese fir changed during tree growth. The bacterial community was influenced by nutrient supply and competition between individual trees. Many secondary metabolites were detectable at high concentrations only at the sapling stage. Phyllosphere bacteria provided various secondary metabolites, such as flavonoids, to Chinese fir saplings and thus promoted sapling growth. Understanding the relationships among stand age, the phyllosphere bacterial community and metabolic profiles will improve our knowledge of the influence of stand age structure on forest functions. The overall expression of genes related to secondary metabolism was substantially different in different stand ages of Chinese fir.

## Data Availability Statement

The original contributions presented in the study are publicly available. This data can be found at: National Center for Biotechnology Information (NCBI) BioProject database under accession number SRR14812903–SRR14812932 under bioproject number PRJNA737303.

## Author Contributions

KS analyzed the data and drafted the manuscript. HS designed the study and supervised the work throughout the research project. ZQ and QL contributed to the installation of the sampling plots and prepared the data. All authors participated in the review and approved the final manuscript.

## Funding

This research was funded by the National Key Technologies Research and Development Program of China (2016YFD0600302-2).

## Conflict of Interest

The authors declare that the research was conducted in the absence of any commercial or financial relationships that could be construed as a potential conflict of interest.

## Publisher's Note

All claims expressed in this article are solely those of the authors and do not necessarily represent those of their affiliated organizations, or those of the publisher, the editors and the reviewers. Any product that may be evaluated in this article, or claim that may be made by its manufacturer, is not guaranteed or endorsed by the publisher.
