# Supplementary material for: Comparative Analyses of Phyllosphere Bacterial Communities and Metabolomes in Newly Developed Needles of Cunninghamia lanceolata (Lamb.) Hook. at Four Stages of Stand Growth
Source: Front Plant Sci. 2021 Sep 28;12:717643. doi: 10.3389/fpls.2021.717643 (PMC8505725; doi:10.3389/fpls.2021.717643)
Supplement: Supplementary file 5 [file Data_Sheet_5.docx]

**Figure S1.** Bacterial species abundance in the phyllosphere of Chinese fir.


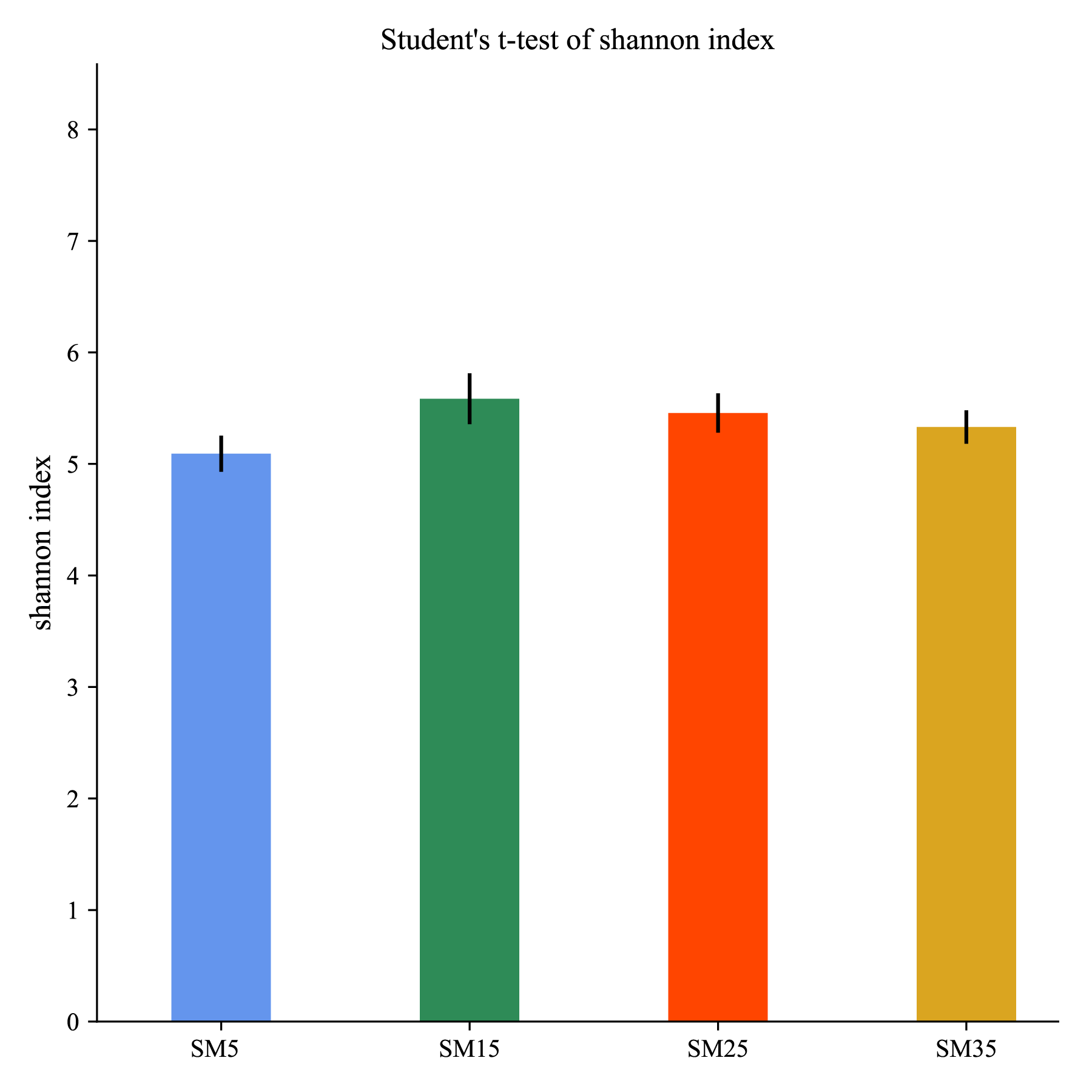

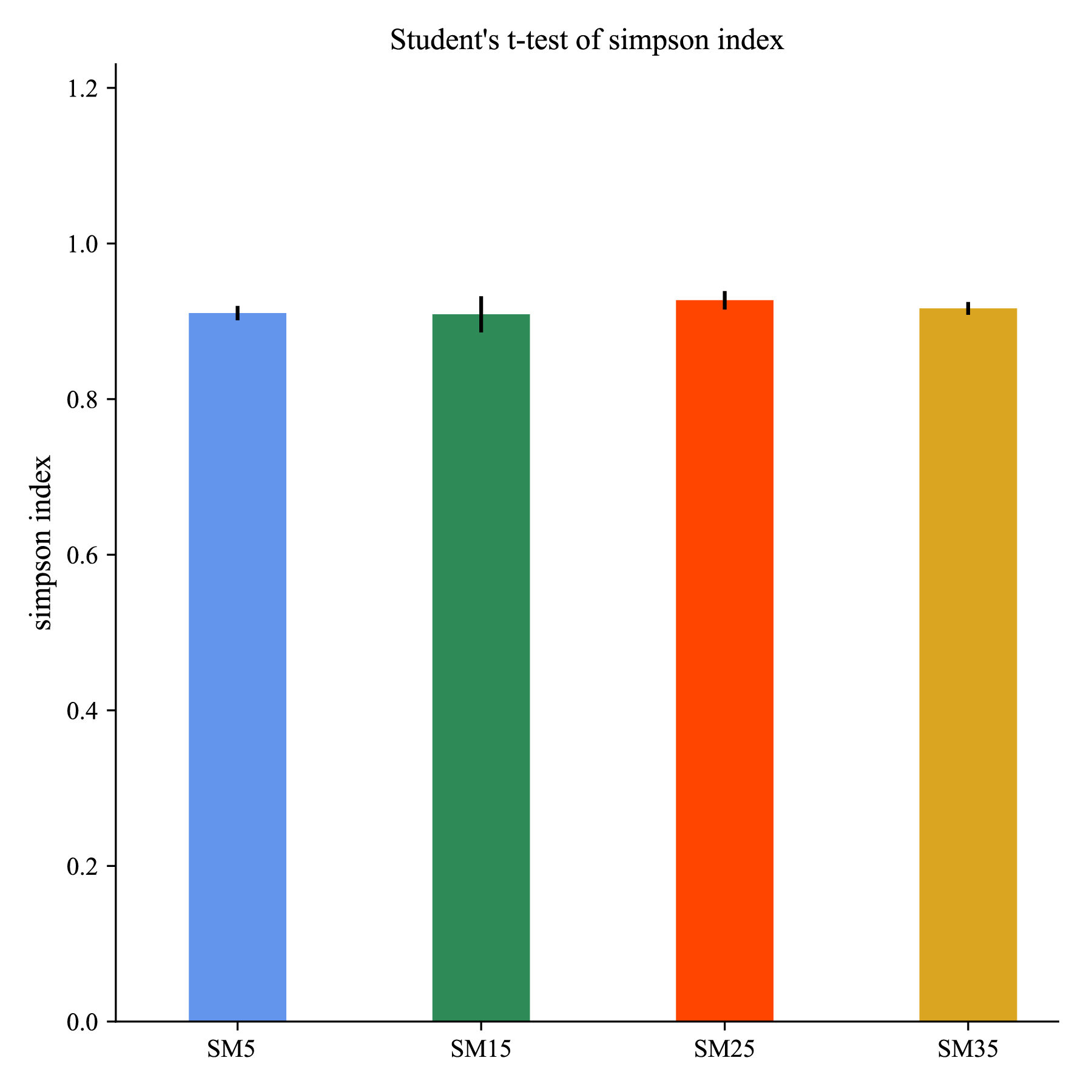


B

C

D

A


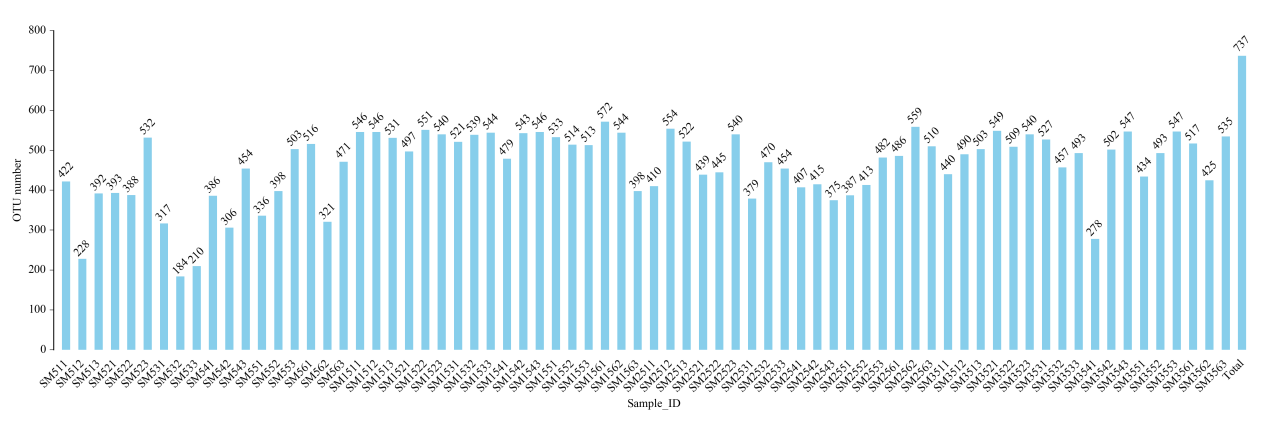

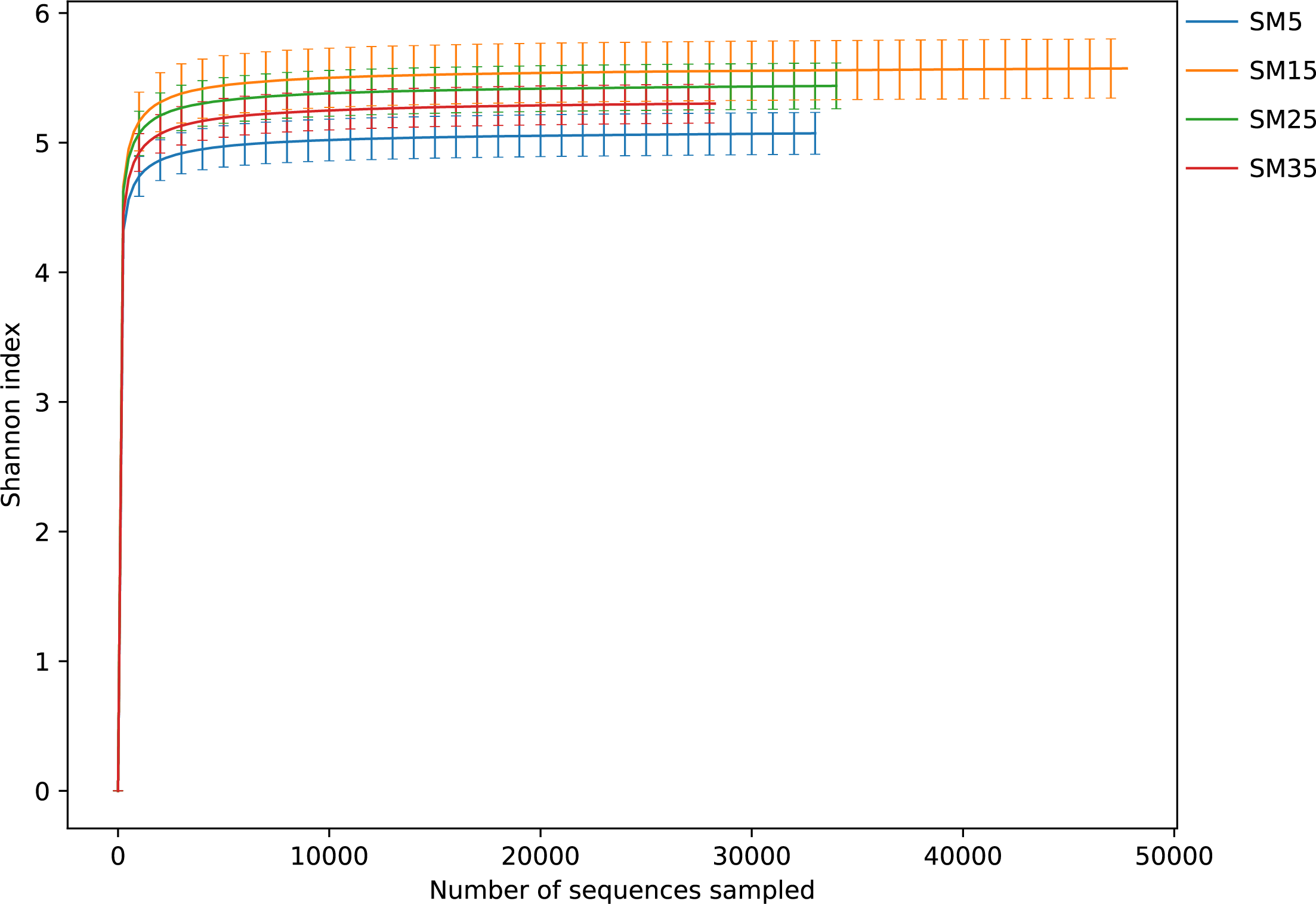


**A**: Number of operational taxonomic units (OTUs) in all samples. **B**: Shannon index for each stand age. **C**: Simpson index for each stand age. **D**: Increase in Shannon index with increasing sequencing depth. SM5, SM15, SM25, and SM35 represent stands of age 5, 15, 25, and 35 years, respectively.

**Figure S2.** Phyllosphere bacterial communities with relative abundance > 0.8% as classified to phylum (**A**), class (**B**), and order (**C**) taxonomic levels. SM5, SM15, SM25, and SM35 represent stands of age 5, 15, 25, and 35 years, respectively.

B

A


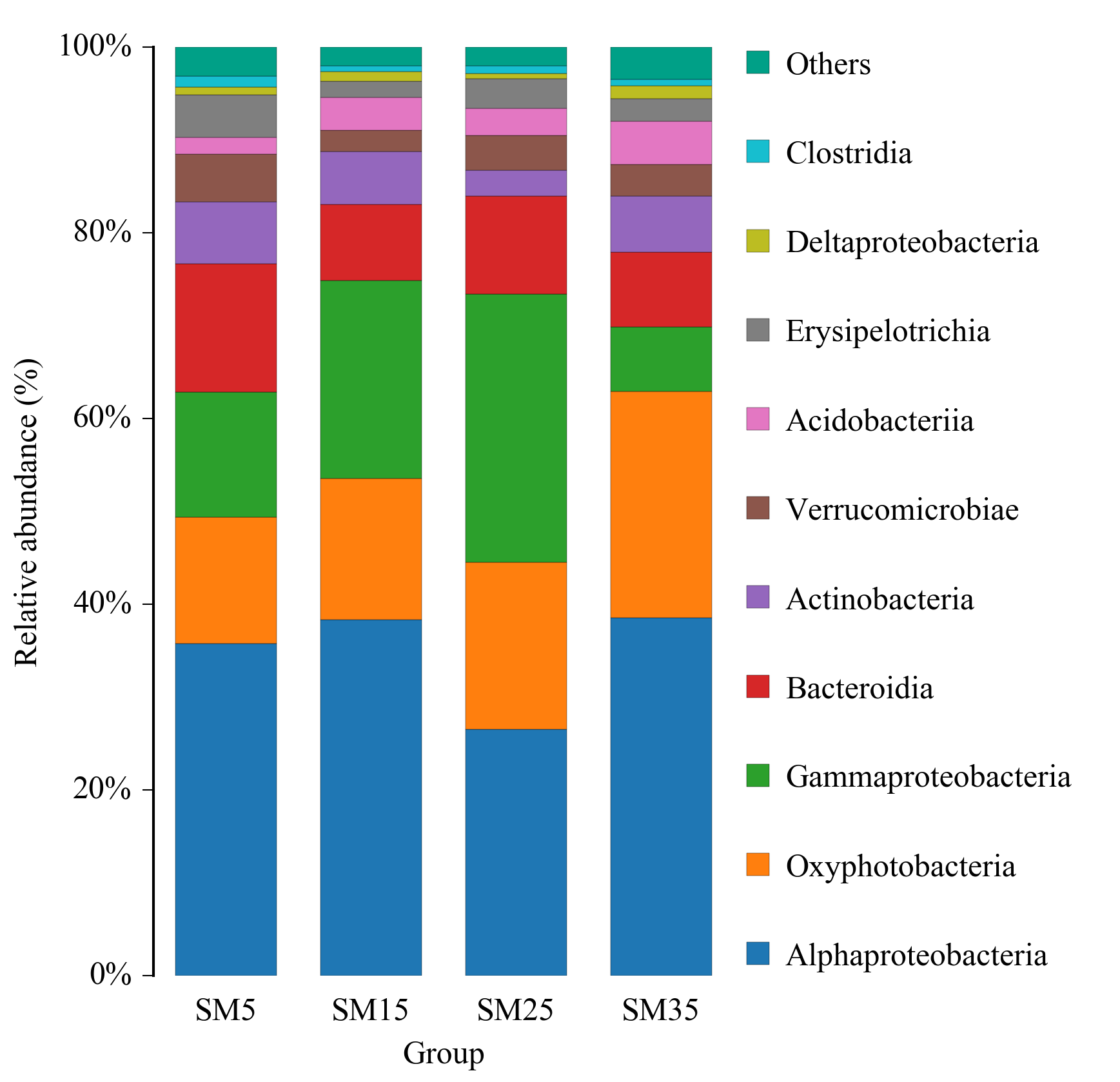

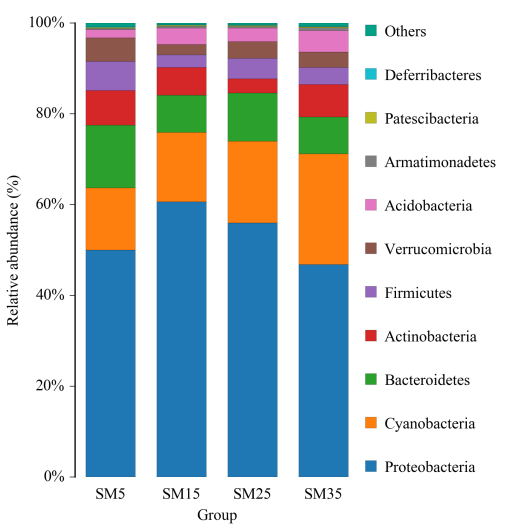

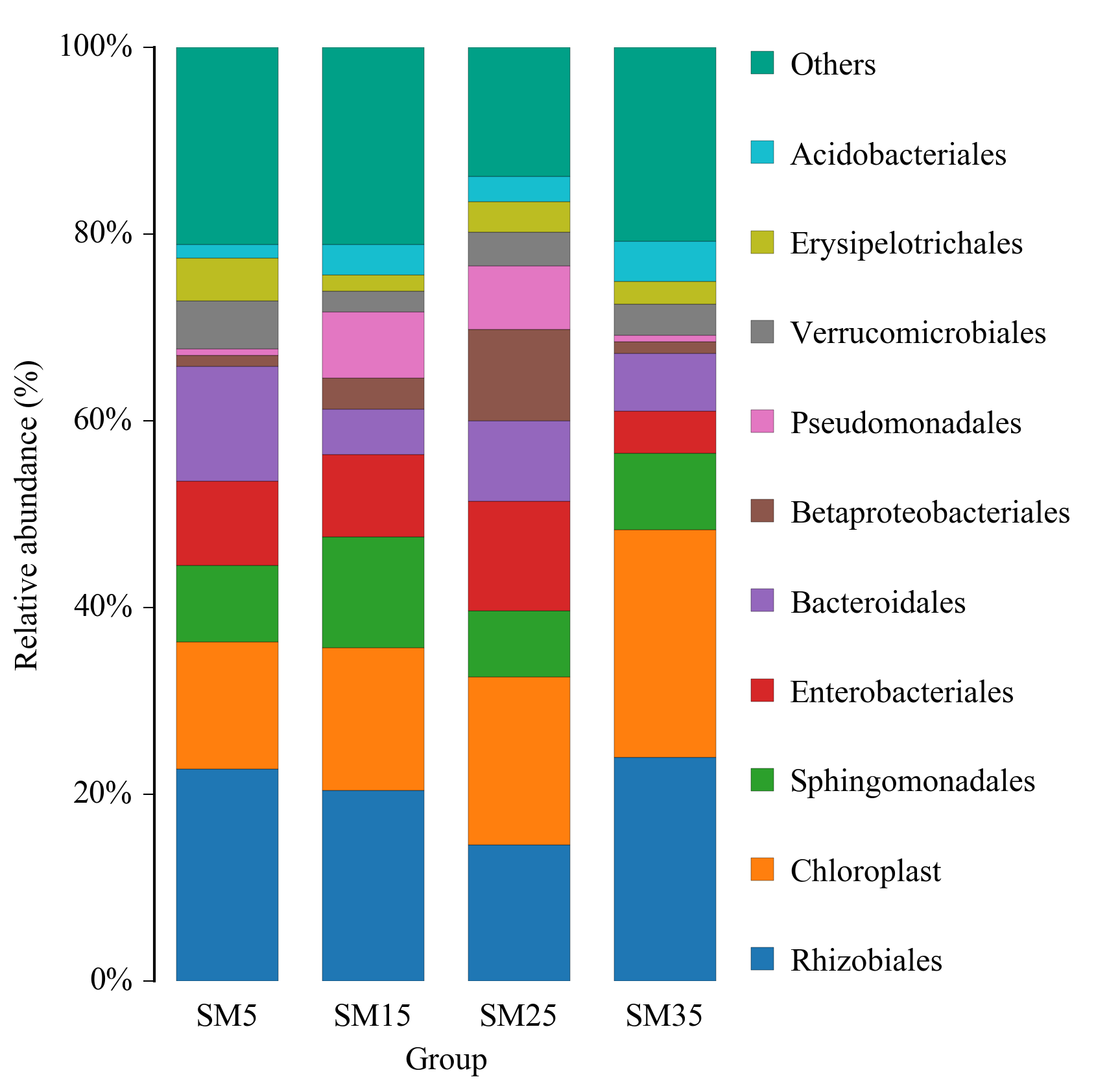


C

**Figure S3.** LEfSe analysis of bacterial taxa showing significant differences in relative abundance among Chinese fir stands of different ages.

C

B

A


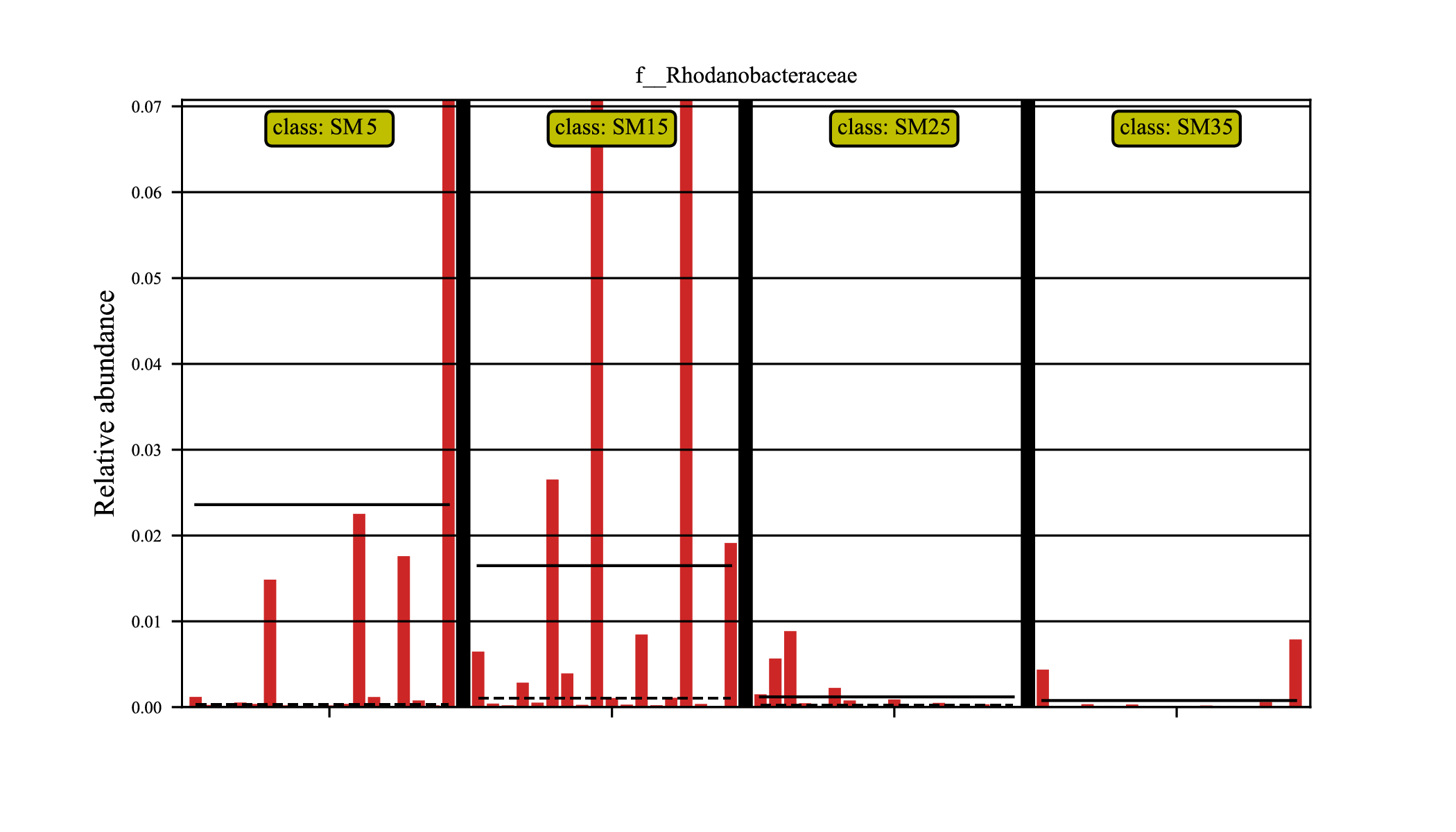

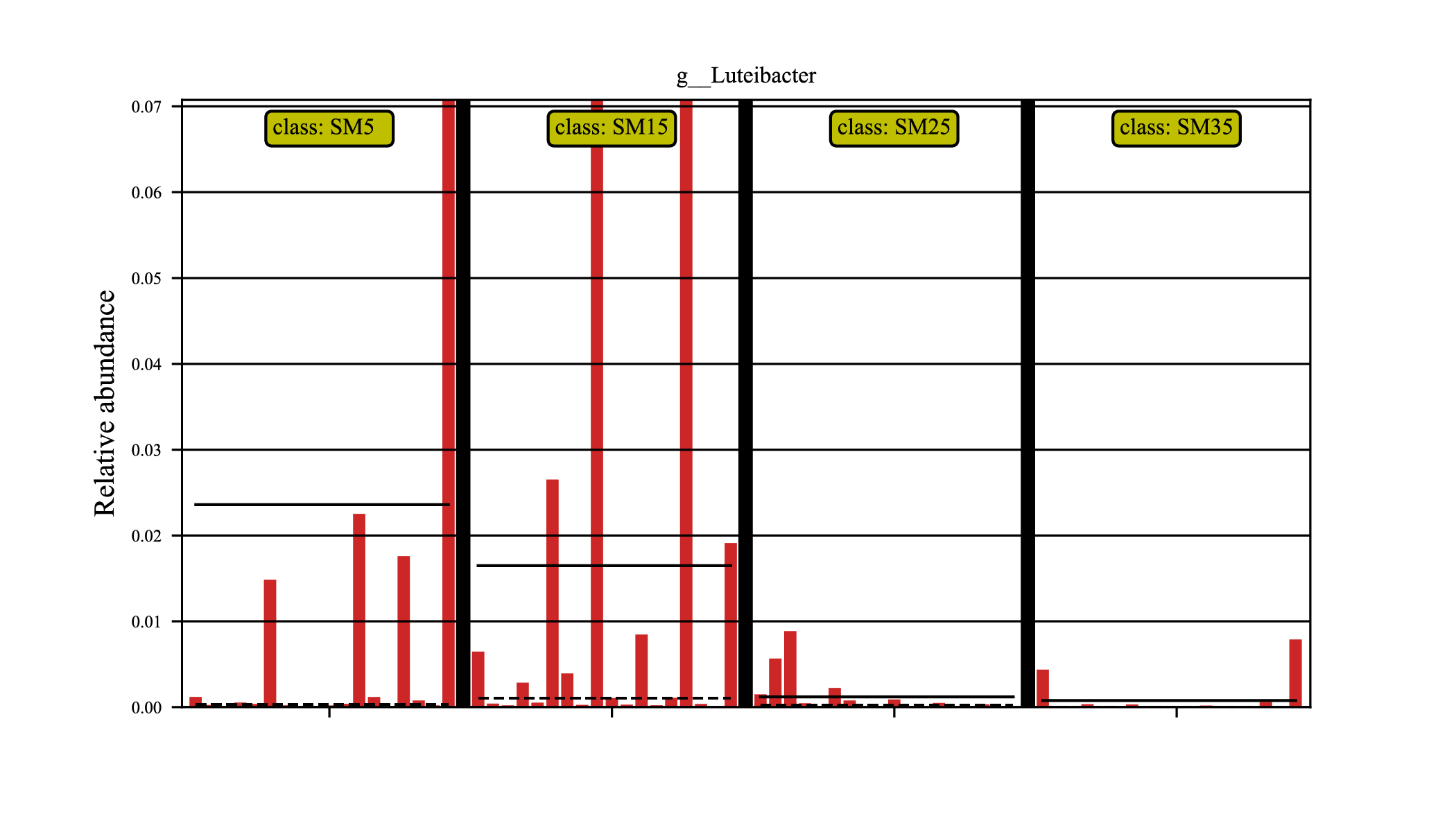

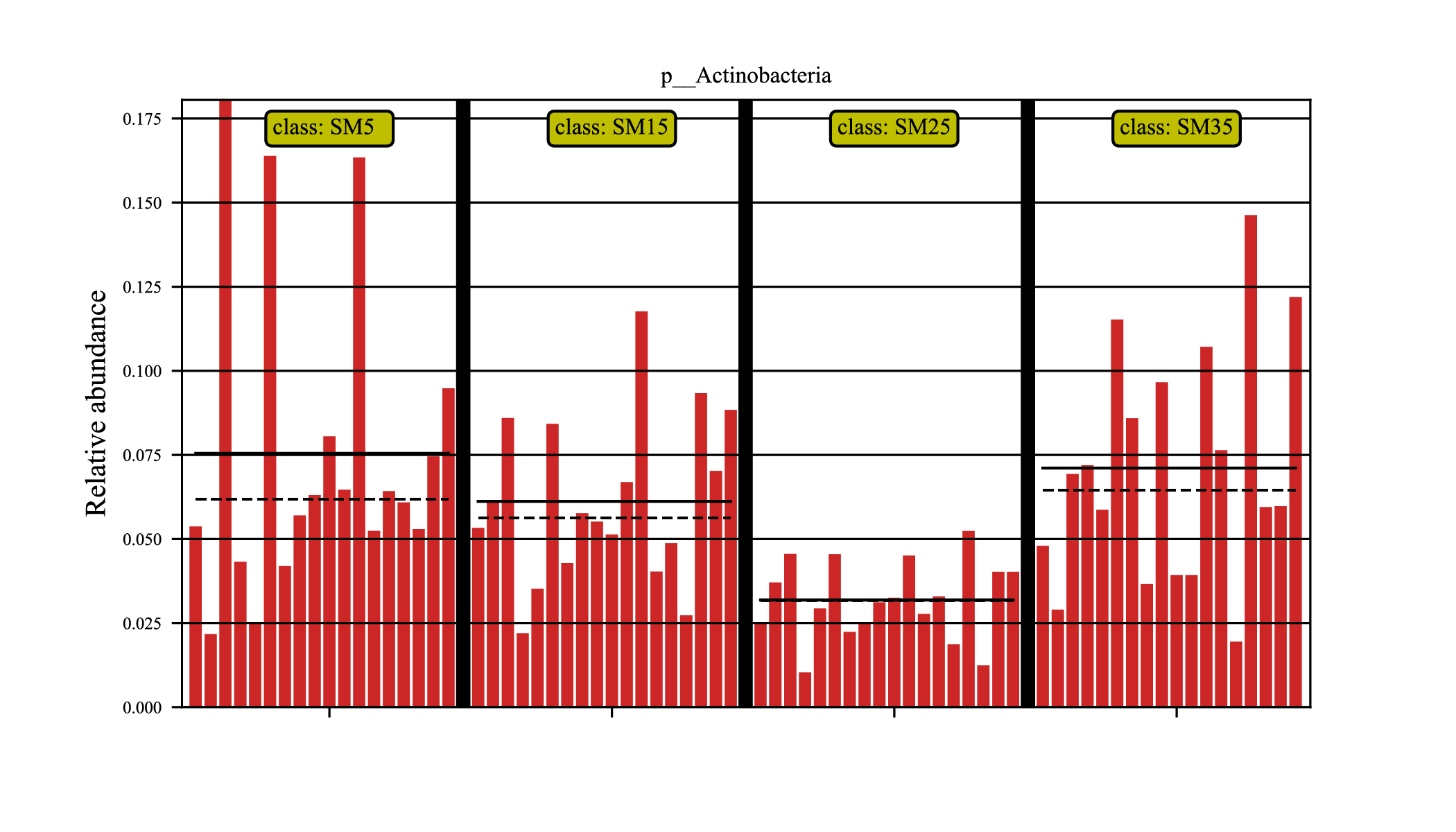


**A**: Rhodanobacteraceae. **B**: *Luteibacter*. **C**: Actinobacteria. SM5, SM15, SM25, and SM35 represent stands of age 5, 15, 25, and 35 years, respectively.

**Figure S4.** Mean proportion of phyllosphere bacterial communities involved in the subfunctions of metabolic pathways that differed significantly in pairwise comparisons of Chinese fir stands of different ages. Pathways were predicted using the KEGG database. SM5, SM15, SM25, and SM35 represent stands of age 5, 15, 25, and 35 years, respectively. **A**: SM5 vs SM25. **B**: SM5 vs SM35. **C**: SM15 vs SM35.


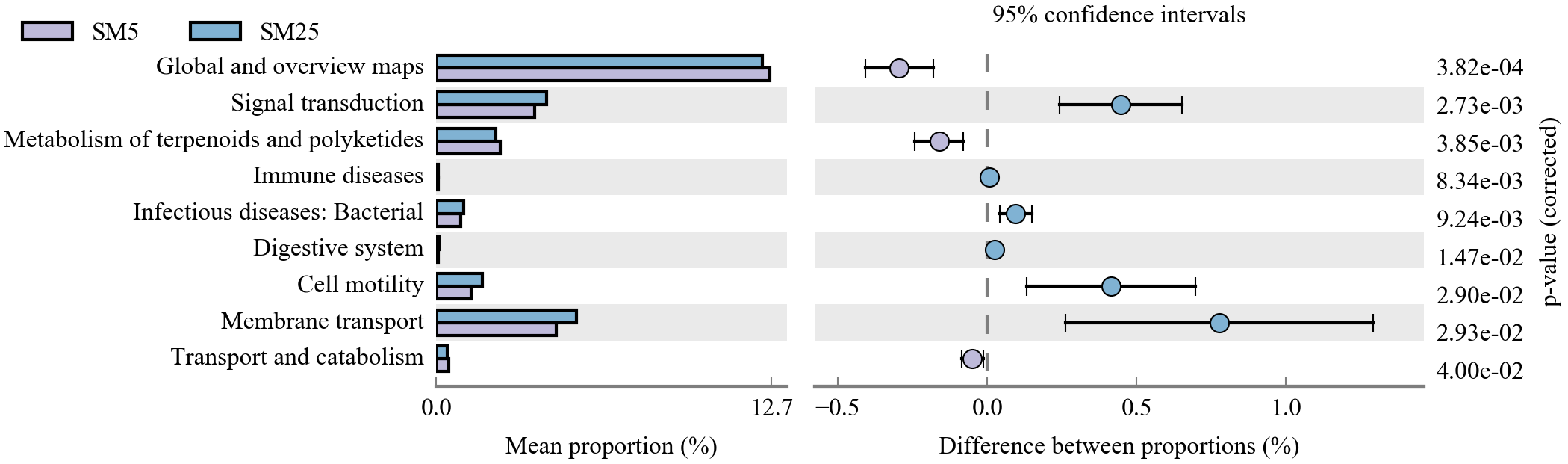


A


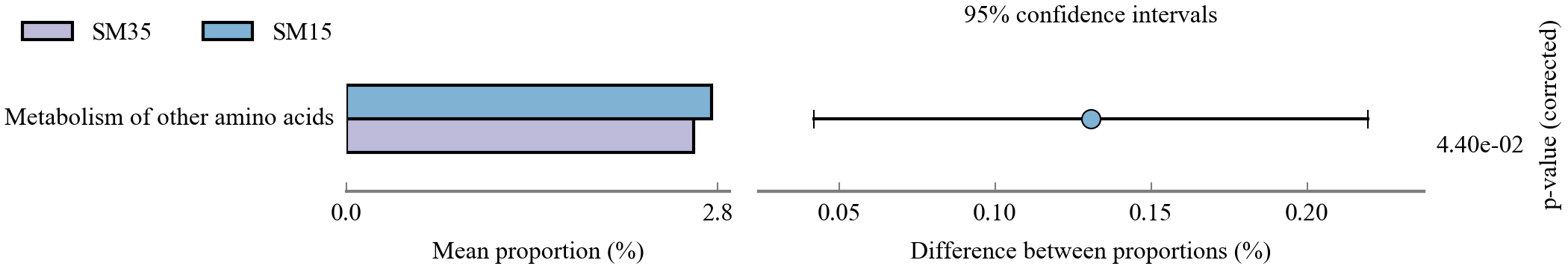


C


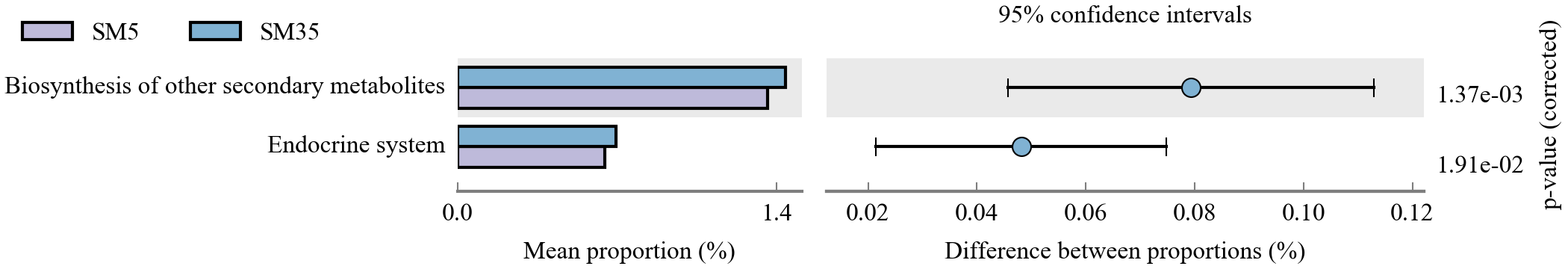


B


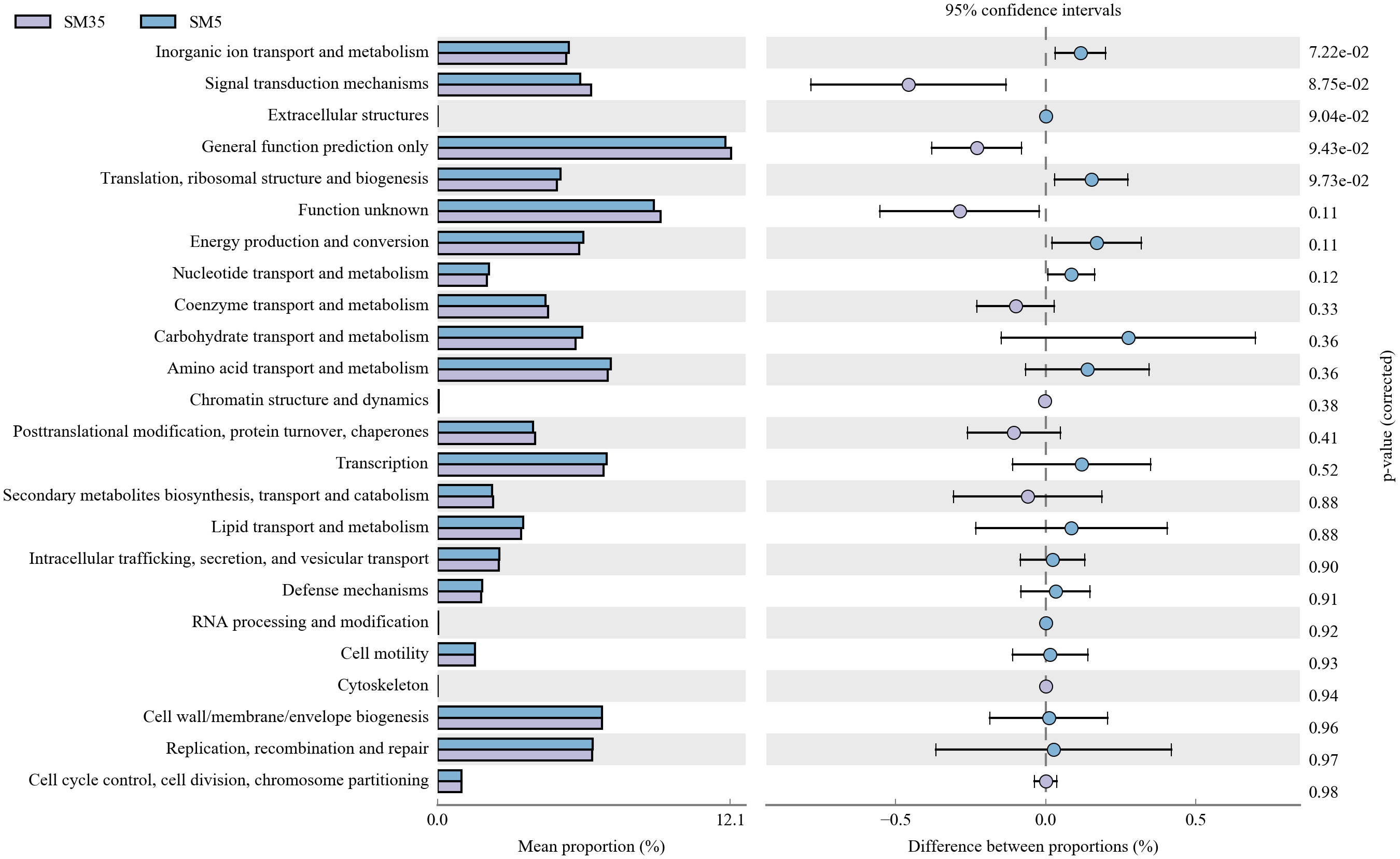


B


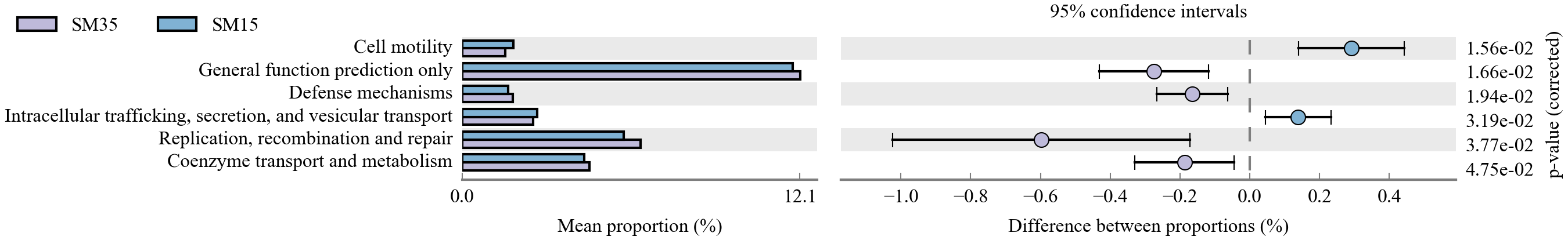


C


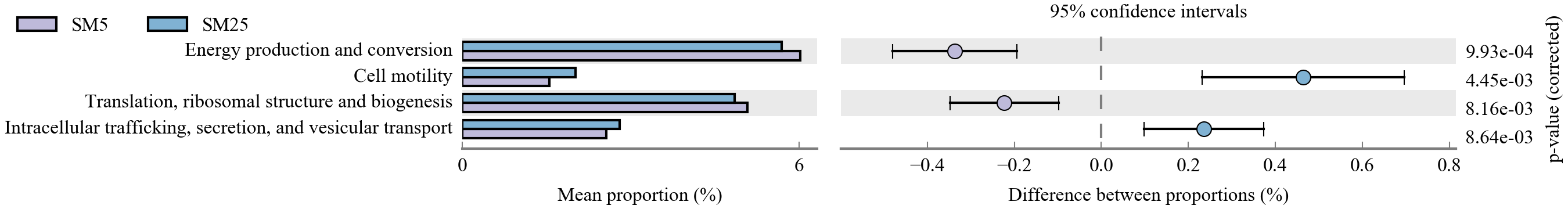


A

**Figure S5.** Mean proportion of phyllosphere bacterial communities of Chinese fir involved in the subfunctions of proteins based on a Clusters of Orthologous Groups analysis. SM5, SM15, SM25, and SM35 represent stands of age 5, 15, 25, and 35 years, respectively. **A**: SM5 vs SM25. **B**: SM5 vs SM35. **C**: SM15 vs SM35.

Figure S6. Metabolites that showed a significant change between any two stands of Chinese fir as indicated by partial least-squares discriminant analysis. 1**5/5**: SM15 vs SM5. **25/5**: SM25 vs SM5. **25/5**: SM25 vs SM5; **35/5**: SM35 vs SM5; **35/25**: SM35 vs SM25; **35/5**: SM35/SM5. SM5, SM15, SM25, and SM35 represent stands of age 5, 15, 25, and 35 years, respectively. Metabolites satisfied the following conditions in pairwise comparisons: (1) ratio ≥ 2 or ratio ≤ 0.5; (2) *q* value < 0.05; (3) VIP ≥ 1; (4) annotation with a secondary metabolite. c: Level of metabolites that significantly changed at least four times in six pairwise comparisons.

**Figure S7.** **Transcript levels of genes encoding enzymes involved in flavonoids biosynthesis based on RNA sequencing.** E2.1.1.104, caffeoyl-CoA *O*-methyltransferase (EC:2.1.1.104); CYP75A, flavonoid 3′,5′-hydroxylase (EC:1.14.13.88); DFR, bifunctional dihydroflavonol 4-reductase/flavanone 4-reductase (EC:1.1.1.219 and EC:1.1.1.234); CYP73A, *trans*-cinnamate 4-monooxygenase (EC:1.14.13.11); LAR, leucoanthocyanidin reductase (EC:1.17.1.3); HCT, shikimate *O*-hydroxycinnamoyltransferase (EC:2.3.1.133); FLS, flavonol synthase (EC:1.14.11.23); CYP75B1, flavonoid 3′-monooxygenase (EC:1.14.13.21); F3H, naringenin 3-dioxygenase (EC:1.14.11.9); CHS, chalcone synthase (EC:2.3.1.74); ANS, leucoanthocyanidin dioxygenase (EC:1.14.11.19); CYP98A, coumaroylquinate (coumaroylshikimate) 3′-monooxygenase (EC:1.14.13.36); ANR, anthocyanidin reductase (EC:1.3.1.77); E5.5.1.6, chalcone isomerase (EC:5.5.1.6). SM5, SM15, SM25, and SM35 represent stands of age 5, 15, 25, and 35 years, respectively.
